# Zero Thermal Expansion and Local Structure in K*_x_* Mn*_x_* Fe_2‐_ *_x_* Mo_3_O_12_‐Based Materials

**DOI:** 10.1002/advs.75304

**Published:** 2026-04-16

**Authors:** Gongsen He, Yongqiang Qiao, Shibo Zhao, Xin Chen, Xiangkai Hao, Kaiyue Zhao, Xinglai Yuan, Mengru Li, Wen Yin, Shintaro Kobayashi, Shogo Kawaguchi, Bingbing Fan, Rui Zhang, Qilong Gao

**Affiliations:** ^1^ School of Materials Science and Engineering Zhengzhou University Zhengzhou China; ^2^ Key Laboratory of Materials Physics Ministry of Education School of Physics Zhengzhou University Zhengzhou China; ^3^ Spallation Neutron Source Science Center Dongguan China; ^4^ Japan Synchrotron Radiation Research Institute (JASRI) Sayo‐cho Hyogo Japan; ^5^ Luoyang Institute of Science and Technology Luoyang China

**Keywords:** local structure, neutron pair distribution function, raman spectroscopy, zero thermal expansion

## Abstract

Zero thermal expansion (ZTE) material is a very interesting research topic for applications. However, achieving ZTE of single‐phase materials over a wide range remains a challenge. In this study, K*_x_* Mn*_x_* Fe_2‐_ *_x_* Mo_3_O_12_ were designed by adjusting the doping ratio of (KMn)^3+^. Local structure can be controlled to regulate thermal expansion. ZTE over a wide temperature range was achieved in the KMnFeMo_3_O_12_ sample (*α_l_* = 0.51 × 10^−6^ K^−1^, 100–800 K). A joint study of synchrotron X‐ray diffraction, neutron pair distribution function, density functional theory calculations, and Raman spectroscopy has been conducted to investigate the thermal expansion mechanism. Even though the macroscopic crystallographic structure of K*_x_* Mn*_x_* Fe_2‐_ *_x_* Mo_3_O_12_ (*x* = 0.4–1.0) adheres to the hexagonal system (*R‐*3*c*) according to the results of synchrotron X‐ray diffraction, the local structure exhibits a monoclinic distortion in K_0.4_Mn_0.4_Fe_1.6_Mo_3_O_12_. In KMnFeMo_3_O_12_, the local structure still exhibits a hexagonal system. This local distortion affects the coupled rotation of polyhedra and leads to different thermal expansion behaviors. Notably, the KMnFeMo_3_O_12_ exhibits low emissivity in the 1–2.5 µm near‐infrared band. This dual functionality, which includes tunable thermal expansion and near‐infrared (NIR) low emissivity, endows the material with considerable potential for advanced engineering applications.

## Graphical abstract

Local structure engineering via ion insertion drives local structural transformation from low‐symmetry P21/a to high‐symmetry R‐3c, enhancing structural flexibility and realizing a transition from positive thermal expansion to wide‐temperature‐range zero thermal expansion in K*_x_* Mn*_x_* Fe_2‐_ *_x_* Mo_3_O_12_‐based materials. This strategy offers a reliable pathway for the rational design of high‐performance zero thermal expansion materials for advanced thermal management applications.

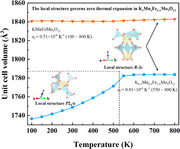

## 1 Introduction

Due to the inherent anharmonicity of the bond potentials, most materials exhibit positive thermal expansion (PTE) [1, 2]. However, in the fields of luminescence, solid oxide fuel cells, aerospace, microelectronic packaging, and high‐precision mechanical systems, the size mismatch caused by thermal expansion will lead to problems such as interface stress and device failure, which restricts the reliability of materials in extreme temperature environments [3, 4, 5, 6, 7]. Negative thermal expansion (NTE) exhibits unusual thermal contraction and cold expansion, which can compensate for PTE to regulate the thermal expansion performance of the material and even obtain a zero thermal expansion (ZTE) material. So far, most of the NTE materials are mainly divided into two categories: one is induced by electron contribution, such as anti‐perovskite manganese nitride [8, 9], Invar‐type alloy [10, 11, 12, 13], intermetal charge transfer (BiNiO_3_, LaCu_3_Fe_4_O_12_) [14, 15], Jahn–Teller effect (*α*‐Cu_2_V_2_O_7_) [16], and spontaneous volume ferroelectric contraction in PbTiO_3_‐based materials [17, 18]. Another is oxides [19, 20, 21], fluorides [22, 23, 24], cyanides [25, 26, 27], and metal–organic frameworks [28, 29], with a framework structure driven by low‐frequency phonon‐related transverse cooperative vibration and the coupling rotation of polyhedra. Precise regulation coefficients of thermal expansion (CTE) are critical, with chemical substitution emerging as the most widely studied strategy. In the MZrF_6_ system, by introducing different cations at the M site to adjust the flexibility of the metal‐fluorine atom bond, a large CTE control window (*α_l_* = −6.69–+18.23 × 10^−6^ K^−1^) can be obtained [24]. However, not all chemical substitutions can significantly regulate thermal expansion properties. For example, the CTE of ZrW_2_O_8_‐based single‐phase materials only shows a slight change (*α*_V_ = −2.2–−2.6 × 10^−5^ K^−1^) after chemical modification, which is mainly limited by low solubility and few substitution sites [30, 31, 32].

As the classical NTE system, the A_2_M_3_O_12_ family (A = trivalent metal ion, M = Mo or W) consists of MO_4_ tetrahedra and AO_6_ octahedra [20, 33]. Notably, many A_2_M_3_O_12_ materials undergo a reversible phase transition from monoclinic to orthorhombic upon heating, with NTE behavior occurring only in the orthorhombic phase [34, 35, 36]. In addition, the moisture absorption is also one of the challenges [37]. Interestingly, NaZr_2_(PO_4_)_3_ (NZP) type materials, with low thermal expansion behavior, exhibit excellent hydrophobicity and thermal stability without thermal‐induced phase transitions, ensuring reliable thermal performance across wide temperature ranges [38]. By combining the compositional flexibility of A_2_M_3_O_12_ with the structural robustness of NZP, a series of novel ABCM_3_O_12_ compounds (A^+^, B^2+^, and C^3+^) has been developed. For instance, in Sc_2_W_3_O_12_, partial substitution of Sc^3+^ with (KMg)^3+^ enhances the NTE performance [39]. A similar trend was also observed in the (KCo)*_x_* Sc_2‐_ *_x_* Mo_3_O_12_ system [40]. Our group also investigated the effects of ion substitution at A, B, C, and M sites on thermal expansion, and designed a new material of RbMnLuMo_3_O_12_, which exhibits a linear contraction (*α*_V_ = −5.28 × 10^−6^ K^−1^, 300–700 K) [41]. Recently, an unusual thermal expansion behavior (positive followed by negative) was observed at *x* = 0.4 in the K*_x_* Mn*_x_* In_2‐_ *_x_* Mo_3_O_12_ series, which is similar to the behavior of the In_2_Mo_3_O_12_ [42]. This phenomenon can be readily correlated with the phase transition behavior of In_2_Mo_3_O_12_. But variable temperature X‐ray diffraction (XRD) analysis indicates that the hexagonal structure is retained throughout the measured temperature range. This observation strongly suggests that local structural changes that cannot be detected by conventional XRD may have occurred. Further investigation into the evolution of the local structure and its impact on macroscopic thermal expansion is therefore essential for both fundamental understanding and practical applications.

Here, Fe_2_Mo_3_O_12_ is selected as the matrix, which undergoes a phase transition from the monoclinic phase to the orthorhombic phase. A series of K*_x_* Mn*_x_* Fe_2‐_ *_x_* Mo_3_O_12_ compounds (*x* = 0–1.1) were synthesized via (KMn)^3+^ co‐substitution in Fe_2_Mo_3_O_12_. Four compositions (*x* = 0.4, 0.6, 0.8, 1.0, denoted as KMn4, KMn6, KMn8, KMn10) were selected for systematic analysis by adjusting the doping ratio of (KMn)^3+^ ions to control the local structure. A joint study of synchrotron X‐ray diffraction (SXRD), neutron pair distribution function (NPDF), density functional theory (DFT) calculations, and Raman spectroscopy was conducted to reveal the connection between local structure and thermal expansion. The specific experimental details and scientific rationales are as follows: temperature‐dependent SXRD measurements were performed on KMn4, KMn6, KMn8, and KMn10 over the range of 100–800 K to precisely determine their long‐range structural evolution, enabling accurate quantification of structure transition temperatures and CTEs. NPDF analysis was carried out on KMn4 and KMn10 to identify potential local structural distortions that may not be detectable by conventional diffraction methods. DFT calculations and Raman spectroscopy further confirmed the existence of the local structure and its correlation with thermal expansion. In addition, the characterization of near‐infrared (NIR) and Mid‐infrared (MIR) emissivity is used to explore the optical performance of KMn10.

## 2 Results and Discussion

### 2.1 Crystal Structure and Thermal Expansion

**FIGURE 1:**
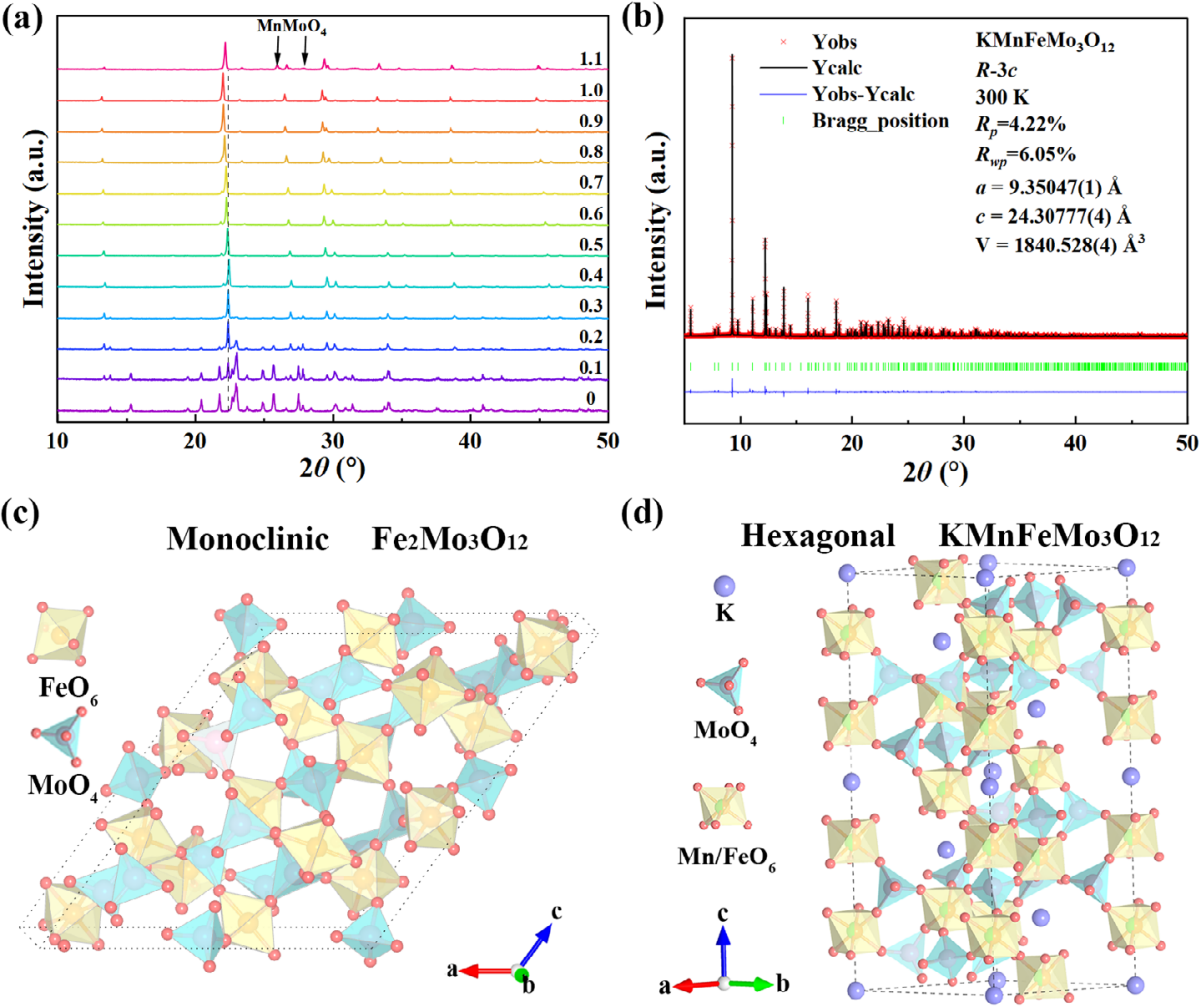
(a) XRD patterns of K*_x_* Mn*_x_* Fe_2‐_ *_x_* Mo_3_O_12_ (*x* = 0.0–1.1), the arrows indicate the diffraction peaks of MnMoO_4_. (b) Rietveld refinement of the SXRD pattern of KMn10 at 300 K. (c) Crystal structure of Fe_2_Mo_3_O_12_. (d) Crystal structure of KMn10.

A series of Fe_2_Mo_3_O_12_‐based compounds, i.e., K*_x_* Mn*_x_* Fe_2‐_ *_x_* Mo_3_O_12_ (0 ≤ *x* ≤ 1.1), was prepared by a solid‐state reaction method. The XRD patterns of K*_x_* Mn*_x_* Fe_2‐_ *_x_* Mo_3_O_12_ (0 ≤ *x* ≤ 1.1) samples at room temperature are shown in Figure 1a. All samples exhibited good crystallinity. When *x* = 0, the positions and relative intensities of the diffraction peaks correspond well with monoclinic Fe_2_Mo_3_O_12_ (PDF: 04‐007‐2787) (Figure S1). The corresponding crystal structure is illustrated in Figure 1c. With the increase of (KMn)^3+^, the same structure as the hexagonal phase KMnFeMo_3_O_12_ (PDF: 04‐020‐1785) began to appear in the sample (Figure S1). When 0.1 ≤ *x* ≤ 0.3, both monoclinic and hexagonal phases coexist. When 0.4 ≤ *x* ≤ 1.0, the monoclinic phase Fe_2_Mo_3_O_12_ completely disappears, and the XRD patterns show a pure hexagonal phase, indicating that a single‐phase K*_x_* Mn*_x_* Fe_2‐_ *_x_* Mo_3_O_12_ (0.4 ≤ *x* ≤ 1.0) was synthesized. When the doping ratio reaches 1.1, the diffraction peaks of orthorhombic MnMoO_4_ begin to appear, indicating that the solid solubility limit of Mn at the Fe site has been reached. It can be observed that with the incorporation of (KMn)^3+^, the main peak gradually moves to a lower angle, which is mainly because of the smaller ionic radius of Fe^3+^ (0.645 Å) compared to Mn^2+^ (0.83 Å) [43].

To obtain more accurate structural data, SXRD measurements were conducted at ratios of *x* = 0.4, 0.6, 0.8, and 1.0, and the SXRD refinement results at 300 K are shown in Figure 1b (KMn10) and Figure S2 (KMn4, KMn6, and KMn8). For KMn10, *R_p_* = 4.22%, *R_wp_* = 6.05%, indicating that the refinement result is accurate enough. The refined structure is shown in Figure 1d, where Mn^2+^ and Fe^3+^ cations coexist in the same position and are randomly distributed. Mn^2+^ or Fe^3+^ cations form Mn/FeO_6_ octahedrons with six O^2−^ ions, and Mo^6+^ cations form MoO_4_ tetrahedrons with four O^2−^ ions. Two Mn/FeO_6_ octahedra and three MoO_4_ tetrahedra are connected along the *c*‐axis direction through shared oxygen atoms, forming a basic unit referred to as a “lantern”. These “lanterns” form a stable framework structure, and the K^+^ cation is located between the “lanterns”. The optimal Rietveld refinement structure parameters of KMn4, KMn6, KMn8, and KMn10 at 300 K are given in Tables S1–S5. X‐ray Photoelectron Spectroscopy (XPS) results of KMn4, KMn6, KMn8, and KMn10 are shown in Figures S3–S6 and indicate that the valence states of the elements in samples are K^+^ [44], Mn^2+^ [45], Fe^3+^ [46], and Mo^6+^ [47].

**FIGURE 2:**
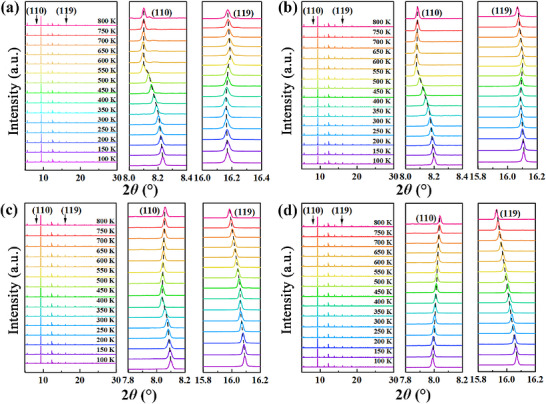
Variable temperature SXRD patterns from 100 to 800 K, and on the right are magnified images of the (110) and (119) diffraction peaks of (a) KMn4, (b) KMn6, (c) KMn8, and (d) KMn10.

To investigate the thermal expansion properties of KMn4, KMn6, KMn8, and KMn10, temperature‐dependent SXRD data in the range of 100–800 K were collected and shown in Figure 2 and Figures S7–S10. Throughout the measured temperature range, no missing or additional diffraction peaks were observed in the SXRD patterns, indicating that no structural phase transition occurred during heating. For KMn4 (Figure 2a), the (110) diffraction peak is significantly shifted to smaller angles between 100 and 550 K. In contrast, the (119) diffraction peak exhibits the opposite trend, suggesting that the lattice parameters along the *a/b*‐axis and *c*‐axis exhibited different trends. This behavior can also be observed in the KMn6 (Figure 2b) and KMn8 samples (Figure 2c), but the transition temperature shifted to 400 K for KMn8. For KMn10 (Figure 2d), both the (110) and (119) diffraction peaks exhibit a monotonic shift with increasing temperature throughout the 100 to 800 K range. This difference is unusual, as all the samples show the same crystal structure within the testing temperature range.

**FIGURE 3:**
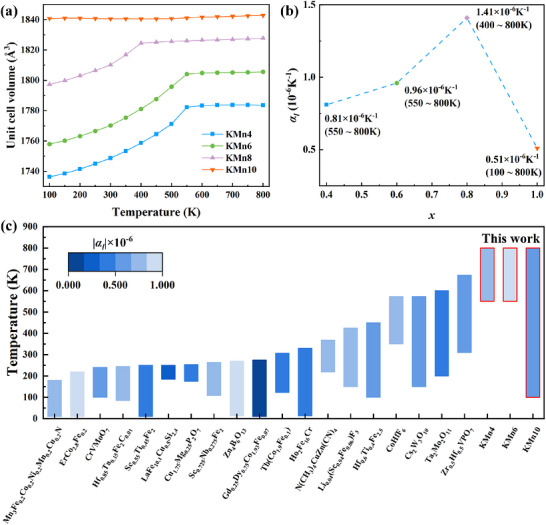
(a) Temperature dependence of unit cell volumes, (b) the variation of linear CTEs with *x* content. (c) Comparison of CTEs and the corresponding temperature windows with conventional ZTE materials.

The lattice parameters of KMn4, KMn6, KMn8, and KMn10 extracted from refined results of SXRD data are shown in Figure S11(*a/b* axis and *c* axis) and Figure 3a (unit cell volume). For the KMn4 and KMn6, the lattice parameters along the *a/b*‐axis increase with temperature between 100 and 550 K, whereas the parameter along the *c*‐axis decreases. The expansion along the *a/b*‐axis is dominant, resulting in PTE of the unit cell volume. In the higher temperature range of 550 to 800 K, the trend reverses: the *a/b*‐axis contracts, while the *c*‐axis expands. As these competing effects are comparable in magnitude, the unit cell volume exhibits ZTE. As the *x* increases, the transition temperature for KMn8 occurs at 400 K, but no turning point is observed for KMn10 across the entire temperature range. And the lattice parameters decrease along the *a/b*‐axis and increase along the *c*‐axis linearly for KMn10, consistent with the trends observed for the (110) and (119) planes (Figure 2). The specific CTE of each axis is listed in Table S6. The linear thermal expansion coefficient *α_l_* is calculated from the bulk thermal expansion coefficient *α*_V_, i.e., *α_l_* ≈ 1/3*α*_V_. As shown in Figure 3b, the CTE after transition temperature for the KMn4, KMn6, and KMn8 are *α_l_* = 0.81 × 10^−6^ K^−1^ (550–800 K), 0.96 × 10^−6^ K^−1^ (550–800 K), and 1.41 × 10^−6^ K^−1^ (400–800 K), respectively. The KMn10 sample does not exhibit any transition across the entire temperature range and achieves a ZTE *α_l_* = 0.51 × 10^−6^ K^−1^ (100–800 K). Compared with typical ZTE materials, the ultra‐wide ZTE temperature window and low CTE of the KMn10 sample show excellent ZTE performance (Figure 3c). The ZTE materials mentioned in the figure are summarized in Table S7, and the references are placed in the Supporting Information.

KMn4, KMn6, and KMn8 exhibit similar thermal expansion properties to Fe_2_Mo_3_O_12_, with PTE at low temperatures and NTE at high temperatures [35]. In Fe_2_Mo_3_O_12_, this transition is induced by a phase transition from monoclinic to orthorhombic. However, all samples exhibit the same structure in the system studied here, whether after the transition temperature. It is speculated that this difference may be due to local structural distortion, which will be further discussed in the following sections.

### 2.2 Local Structure Analysis

By analyzing the variation of lattice parameters with temperature, local structural distortions may exist in KMn4, KMn6, and KMn8, leading to the different thermal expansion behaviors between KMn4 and KMn10. To investigate the local structure, NPDF measurement was conducted on KMn4 and KMn10. Initially, the hexagonal *R‐*3*c* model consistent with the SXRD result was adopted to analyze the NPDF data of KMn4 and KMn10.

**FIGURE 4:**
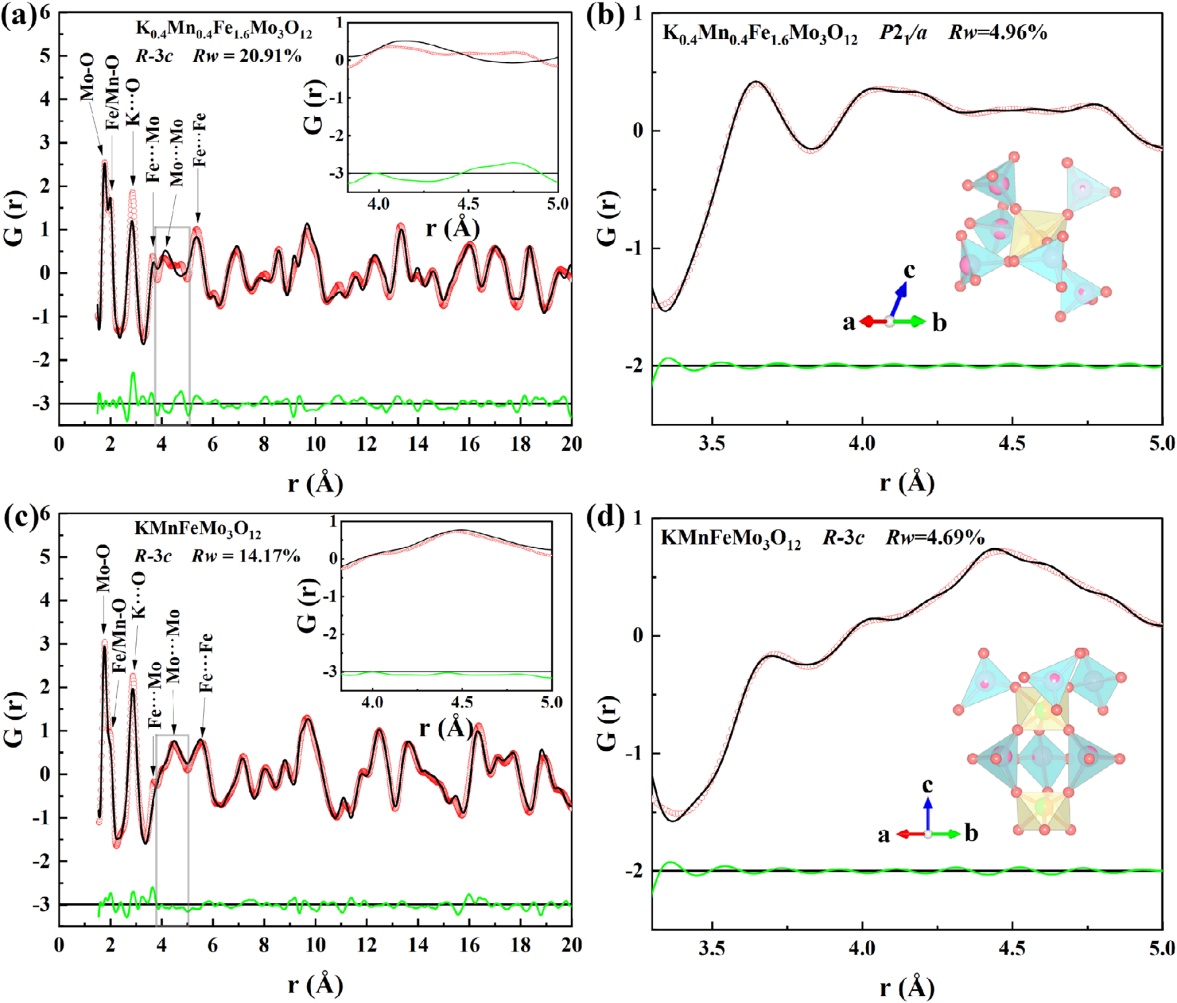
NPDF fitting at 300 K with the *R‐*3*c* model at *r* (1.5–20 Å) for the (a) KMn4, (c) KMn10. The illustrations in Figure 4a, c are enlarged versions of the fitting results within the range of r (3.8–5 Å). (b) NPDF fitting at 300 K with the *P*2_1_ */a* model for the KMn4 at *r* (3.3–5 Å) and (d) with the *R‐*3*c* model for the KMn10 at *r* (3.3–5 Å). Illustrations of Figure 4b, d show the positions of FeO_6_ octahedra and the adjacent MoO_4_ tetrahedra in the monoclinic model and hexagonal model, respectively. The red circles correspond to the experimental data, the black line represents the fitted data, and the difference curves are shown by green lines. NPDF peaks labeled by arrows correspond to different atom pairs.

As shown in Figure 4a, c, the NPDF data of KMn4 and KMn10 in the long‐range region (5–20 Å) can be well fitted using the *R‐*3*c* structural model. This indicates that both samples adopt a hexagonal phase as their average structure, which is consistent with the Rietveld refinement results of the SXRD data. However, in the short‐range region (3.8–5 Å) corresponding to the Mo···Mo atomic pairs, distinct differences emerge in the NPDF peak shapes for the two samples. The experimental data of KMn10 were well fitted by the hexagonal phase model (the inset in Figure 4c). In contrast, KMn4 exhibits obvious peak splitting that cannot be fitted well (the inset in Figure 4a). This peak‐splitting phenomenon reveals multiple distinct Mo···Mo distances in the local structure of KMn4, which deviates from the ideal hexagonal symmetry [48]. Consequently, KMn4 shows poorer overall fitting quality compared to KMn10 (KMn4: *R_w_* = 20.91%; KMn10: *R_w_* = 14.17%).

Given that the matrix Fe_2_Mo_3_O_12_ belongs to the monoclinic system (space group *P*2_1_ */a*), the monoclinic mode (*P*2_1_ */a*) was used to describe the possible local structure of KMn4 at low r range (3.3–5 Å). As shown in Figure 4b, refinement based on the *P*2_1_ */a* model yields a considerably improved fitting of experimental data (*R_w_* = 4.96%), suggesting that the local structure of KMn4 is predominantly monoclinic. Similarly, the symmetry of the local structure is lower than that of the average structure, which has also been reported in other materials, such as (Sc_0.85_Ga_0.05_Fe_0.1_)F_3_ [49], Li_2_SrSiO_4_ [50], and AgGaTe_2_ [51]. In contrast, the *R‐*3*c* model provides an excellent fit for KMn10 within the same short‐range region (*R_w_* = 4.69%, Figure 4d), indicating that the local and average structures of KMn10 are consistent with the hexagonal phase.

The local structure schematics (the insets in Figure 4b, d) were constructed based on the fitting results, intuitively reflecting the discrepancy in atomic arrangement between the monoclinic and hexagonal phases. For the local monoclinic structure of KMn4 (the inset in Figure 4b), the arrangement of MoO_4_ tetrahedra around Mn/FeO_6_ octahedra is more disordered, which inhibits the coupled rotation of the polyhedra and accounts for the PTE of KMn4 at low temperatures. In contrast, the hexagonal structure of KMn10 (the inset in Figure 4d) features highly ordered “lantern” units, whose cooperative rotation facilitates the emergence of ZTE behavior. Therefore, the monoclinic distortion in the local structure of KMn4 is responsible for the discrepancy in low‐temperature thermal expansion behaviors between KMn4 and KMn10, and confers a PTE behavior on KMn4 at low temperatures.

**FIGURE 5:**
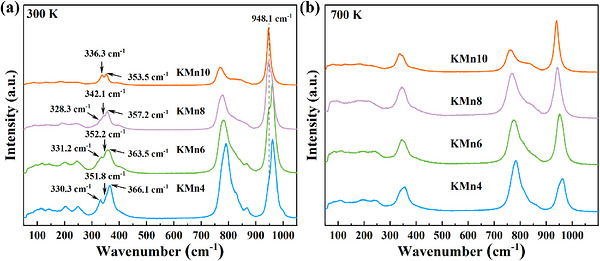
Raman spectra of KMn4, KMn6, KMn8, and KMn10 at (a) 300 K, (b) 700 K. The arrows in (a) represent the transition from three to two Raman peaks.

Phonon vibrations of different chemical bonds exhibit characteristic frequencies, and Raman spectroscopy is highly sensitive to reflecting the vibrational modes of materials [52], which can effectively reflect the change of local structure [53, 54]. To investigate the evolution of the local structure of the materials with composition and temperature, variable‐temperature Raman measurements were performed. As shown in Figure 5a, at 300 K, the number of Raman modes gradually decreased with the increase in Mn content from KMn4 to KMn10. Specifically, in the wavenumber range of 300–400 cm^−1^, the Raman peaks evolved from three modes (for KMn4, KMn6, and KMn8) to two modes; in the high‐wavenumber range of 900–1000 cm^−1^, the double peaks gradually merged into a single peak. Generally, the number of Raman modes is correlated with structural symmetry, with a lower symmetry corresponding to a greater number of vibrational modes [55, 56]. These changes indicate that the local symmetry of the materials is enhanced with the increase in Mn content, which is consistent with the results of PDF analysis. In addition, the Raman spectra at 700 K reveal that the peak shapes of all compositions tend to be consistent in the corresponding wavenumber ranges, all of which are characteristic of high local symmetry (Figure 5b). For instance, all samples exhibit two Raman modes in the range of 300–400 cm^−1^. This demonstrates that thermal perturbation effectively suppresses the local lattice distortion, unifying the structures of different compositions into a high‐symmetry hexagonal phase.

**FIGURE 6:**
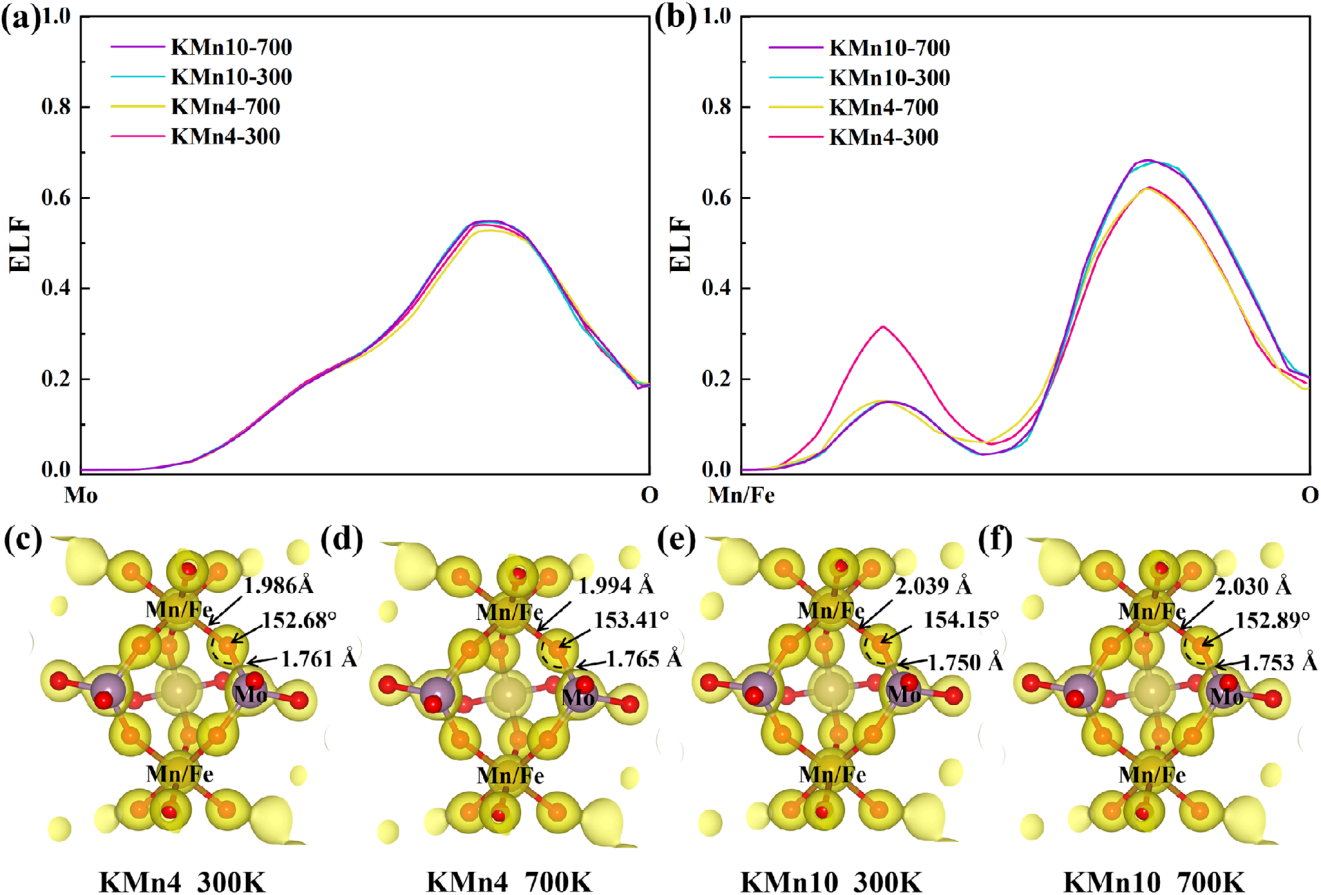
ELF profiles calculated along the (a) Mo‐O, (b) Mn/Fe‐O. Charge density diagrams of (c) KMn4 at 300 K, (d) KMn4 at 700 K, (e) KMn10 at 300 K, and (f) KMn10 at 700 K. The Mo‐O, Mn/Fe─O bond lengths and Fe─O─Mo bond angles are labeled.

After confirming the existence of local monoclinic distortion in KMn4 by NPDF and Raman analysis, the electronic structure of the samples was further explored. The electron localization function (ELF) is highly sensitive to variations in the bonding environment and can provide complementary evidence for local structural distortion from the perspective of electron localization [57]. Therefore, the electronic properties of KMn4 and KMn10 were calculated at 300 K and 700 K, and the results are presented in Figure 6. Changes in the ELF values reflect the nature of chemical bonds and the evolution of the lattice in mechanical behavior [58]. As shown in Figure 6a, the ELF value of Mo─O bonds remains unchanged with temperature and doping ratio, indicating that the MoO_4_ tetrahedra are relatively rigid, which is consistent with the existing research [33]. In contrast, the ELF variations of Mn/Fe─O bonds are more intriguing. The Mn/Fe─O bonds show ionic bond characteristics, and the ELF value of KMn4 at 300 K near Mn/Fe ions is abnormally large, indicating that Mn/Fe ions have a more localized charge in KMn4 at 300 K (Figure 6b), and this corresponds to the local structural distortion of KMn4 at low temperature. Additionally, the degree of electron localization around O ions is enhanced with the doping ratio, which indicates that the Mn/Fe─O bond in KMn10 has stronger ionic bond characteristics. This change in bonding properties directly affects the rigidity of the bond. As shown in Figure 6e, f, KMn10 with stronger ionic bond characteristics exhibits a longer Mn/Fe─O bond length, indicating that the strength of Mn/Fe‐O in KMn10 is weaker and the structure is more flexible. In addition, the Mn/Fe─O─Mo bond angle of KMn10 changes up to 1.26° during heating (Figure 6e, f), while the bond angle in KMn4 changes only 0.73° due to the limitation of local structural distortion (Figure 6c, d).

### 2.3 ZTE Mechanism

**FIGURE 7:**
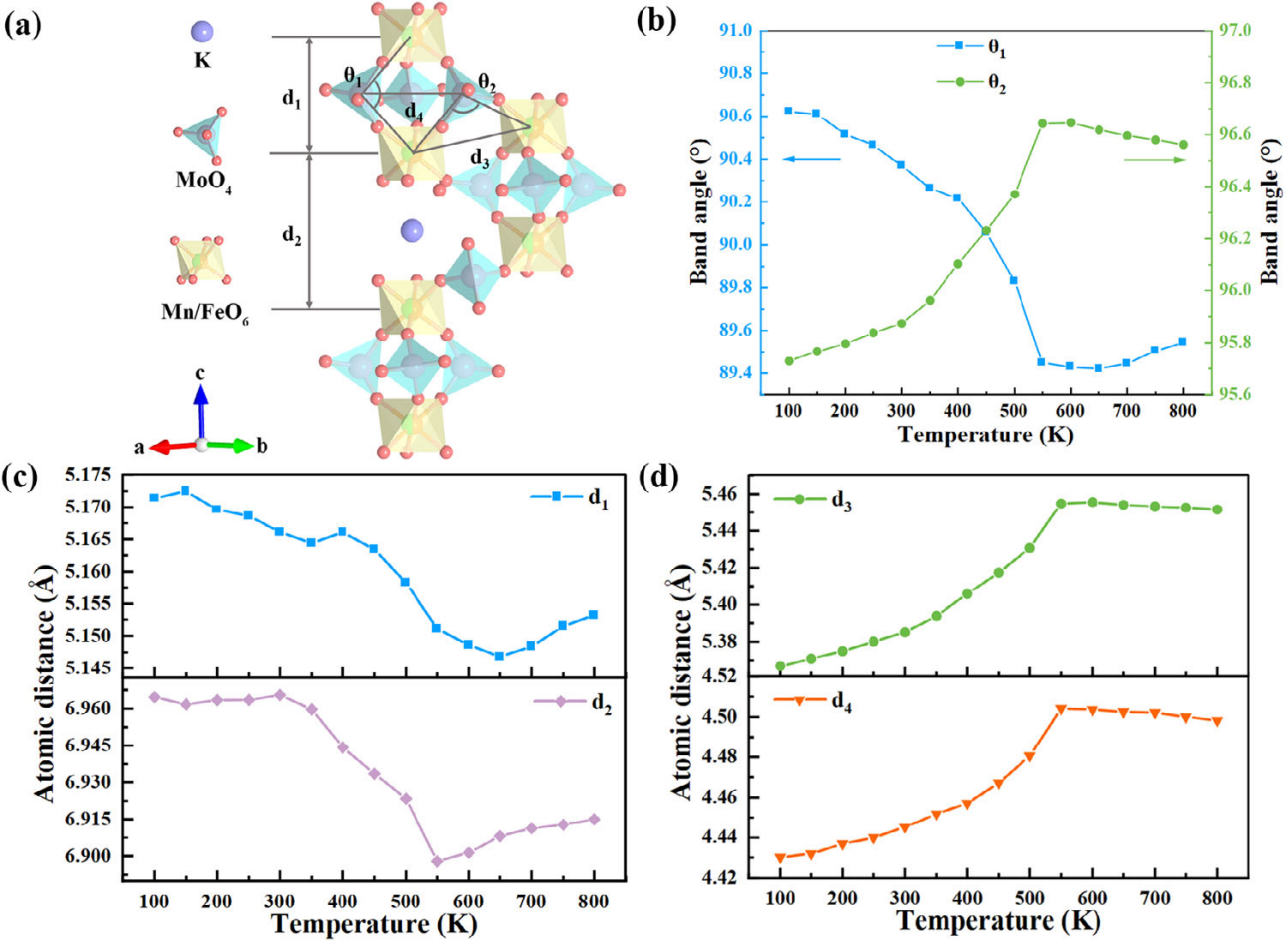
(a) The structure diagram of “lantern” units. (b) Temperature dependence of the non‐bonded angle of θ_1_ and θ_2_ in KMn4. Temperature dependence of the non‐bonded atom distances of (c) d_1_, d_2_, and (d) d_3_, d_4_.

To investigate the distinct thermal expansion behavior of KMn4, the bond distances and angles of KMn4 are extracted from the refinement result based on the SXRD data. Figure 7a illustrates a “lantern” and its neighboring “lantern” along the *a/b‐*axis and *c‐*axis directions. In the *c*‐axis direction, the non‐bonding bond angle θ_1_ decreases first and then increases (Figure 7b), resulting in the distance of d_1_ inside the lantern first decreases and then increases, and d_2_ between the lanterns exhibits similar changes (Figure 7c), which together lead to the first decreases and then the increases of the *c*‐axis direction. In the direction of the *a/b*‐axis, the non‐bonding bond angle θ_2_ increases first and then decreases (Figure 7b), which leads to the distance of d_3_ between adjacent lanterns increases first and then decreases (Figure 7d), and the d_4_ bond length inside the lantern shows the same trend as d_3_, the combined effect of both leads to the expansion and subsequent contraction of the *a/b*‐axis. It is worth noting that the inflection point of all bond lengths and bond angles is near 550 K, which further indicates that there is a change in the local structure. As a result, the anisotropic thermal expansion competition between the *a/b* axis and the *c* axis leads to the low‐temperature PTE and high‐temperature ZTE of KMn4.

**FIGURE 8:**
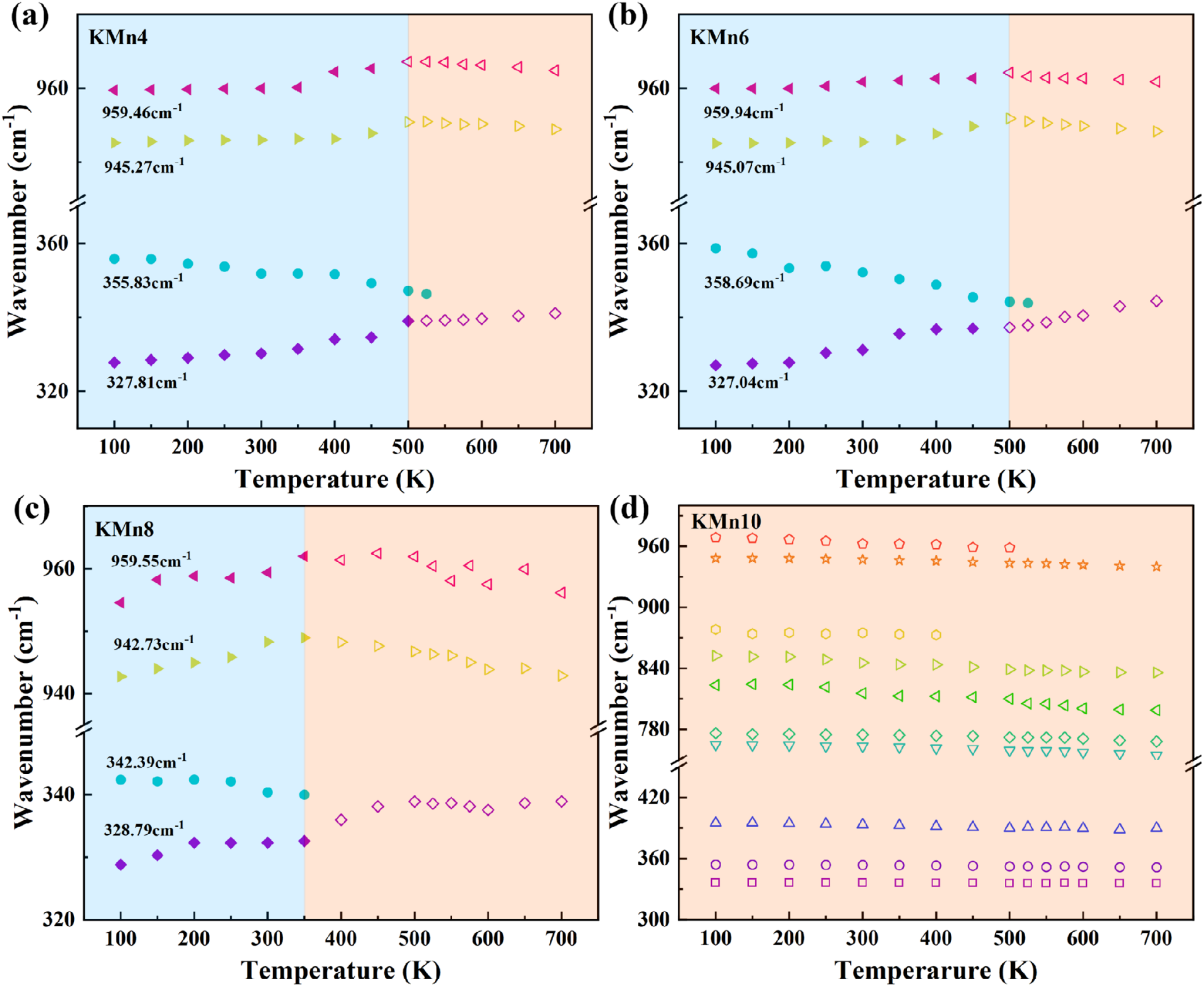
Some anomalous Raman modes with temperature from 100 to 700 K of (a) KMn4, (b) KMn6, (c) KMn8, and (d) KMn10.

Lattice dynamics analysis is used to further elucidate the thermal expansion behavior of KMn4, KMn6, KMn8, and KMn10 via Raman spectroscopy. The temperature‐dependent Raman spectra of KMn4, KMn6, KMn8, and KMn10 are shown in Figure S12. The Raman modes in the 300–400 cm^−1^ range are associated with the bending vibrations of MoO_4_ groups, and those in the 750–1000 cm^−1^ range correspond to the stretching vibrations of MoO_4_ groups [59]. As shown in Figure 8a–c, the Raman modes of KMn4 and KMn6 near 355 cm^−1^ disappear at about 525 K, and the Raman mode at 342.39 cm^−1^ in KMn8 disappears when the temperature is higher than 350 K. In addition, the high‐wavenumber Raman modes near 945 cm^−1^ and 959 cm^−1^ also show abrupt changes near their corresponding structural transition temperatures (Tables S8–S10). These Raman modes usually exhibit softening with increasing temperature (e.g., in KMn10), but KMn4, KMn6, and KMn8 exhibit abnormal hardening at low temperatures, which reduces the overall structural flexibility. These synchronous anomalies of these vibration modes provide direct spectroscopic evidence for the existence of local structural distortion. For KMn4, KMn6, and KMn8, the Raman mode near 327 cm^−1^ (bending vibrations of MoO_4_ groups) displays a continuous blue shift throughout the temperature range, which contributes to NTE. In the low temperature region with local structural distortion, the mode near 355 cm^−1^ exhibits strong red shifts whose effect dominates over the blue shift contribution, resulting in significant PTE. When the temperature exceeds the local structure transition temperature, the red shift effect is weakened, and the blue shift effect persists, resulting in ZTE behavior. In contrast, all Raman modes in KMn10 show consistent slight red shifts across the whole temperature range, as exemplified by modes at 336.15 cm^−1^ and 354.07 cm^−1^ with total anharmonicity values of −0.39 × 10^−5^ K^−1^ and −1.40 × 10^−5^ K^−1^, respectively (Figure 8d; Table S11). This cooperative weak red shift behavior collectively contributes to the ZTE.

For oxide material systems, the torsional and rotational motions of structural units are closely related to the transverse vibration of oxygen atoms. Therefore, the atomic anisotropy displacement parameters (ADPs) are extracted from the temperature‐varying SXRD refinement data. For KMn4 and KMn6 (Figure S13a, b), the results show that the U11 of O1 and the U11 and U22 modes of O2 exhibit non‐monotonic temperature dependence: the values decrease with the increase of temperature at first, and then increase. Interestingly, the inflection point is consistent with the transition point of thermal expansion behavior. This indicates that the transverse vibration of the oxygen atom is suppressed by the local monoclinic structure, which leads to the PTE of KMn4, KMn6, and KMn8. Above 550 K, the local structure is transformed into a hexagonal phase, which eliminates the constraint on oxygen atoms. The transverse vibration of oxygen atoms increases with temperature, which is more conducive to the coupling rotation of polyhedra, thus making the material exhibit ZTE. In KMn8, the inflection point moves to 400 K (Figure S13c). For KMn10, the vibrations of oxygen atoms in all directions increase monotonically with increasing temperature (Figure S13d), indicating that the local structure plays a decisive role in the thermal expansion behavior.

### 2.4 Infrared Emissivity

**FIGURE 9:**
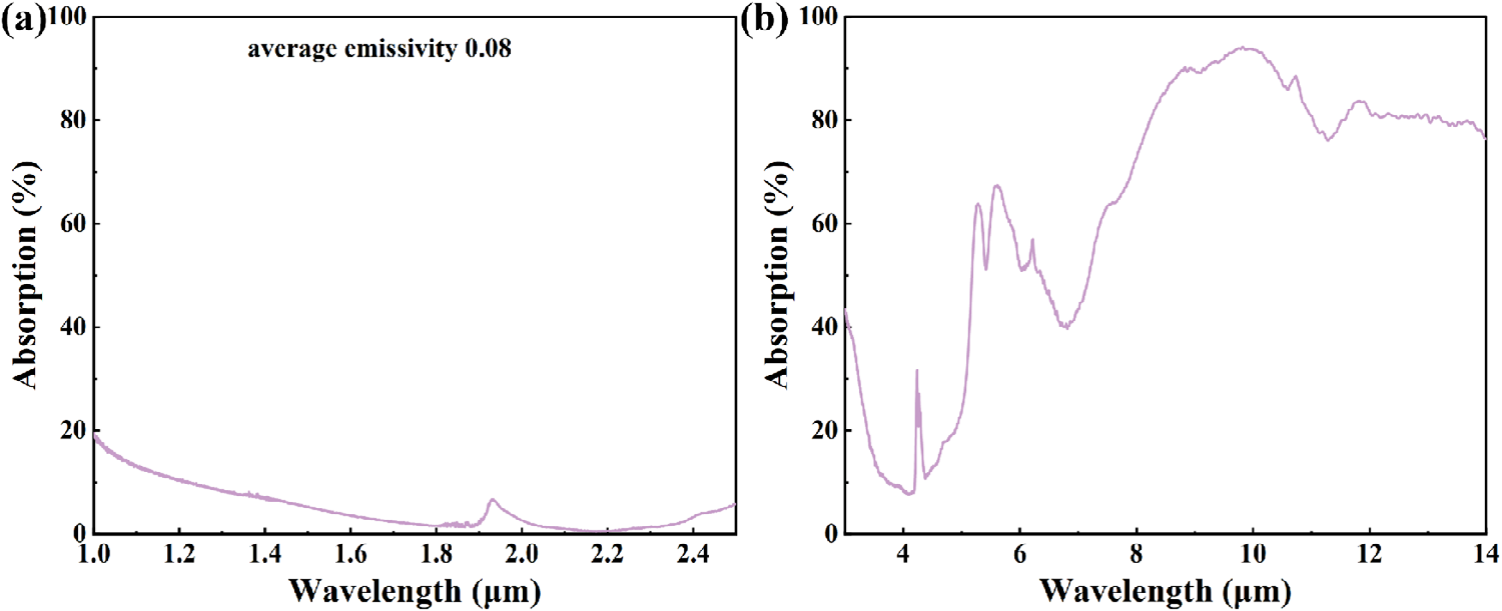
The infrared performance of KMn10: (a) absorptivity spectra in the 1–2.5µm band; (b) emissivity spectra in the 3–14µm band.

The NTE in framework oxides originates from the dynamic motion of structural units, a process that inevitably changes the chemical bonding state and affects the infrared radiation behavior of the material. Given this connection, we characterized the KMn10's emissivity in the near/mid‐infrared bands. Consistent with Kirchhoff's law, the specimen exhibits a low near‐infrared band absorptivity (average ∼ 0.08, Figure 9a), which confers high reflectivity to minimize heat absorption and associated rapid temperature fluctuations [60]. This is effectively complemented by its dual‐response characteristic in the mid‐infrared band, where a high emissivity (8–14 µm, average > 80%, Figure 9b) facilitates efficient heat dissipation, further suppressing thermal deformation caused by heat cycling [61]. The successful integration of these “infrared thermal management” capabilities with inherent “thermal stability” allows the material to meet the demanding requirements of a wide range of application scenarios. This synergistic “thermal expansion‐infrared emission” property unlocks promising application potential for the KMn10 sample in optical, military, and energy sectors.

## 3 Conclusions

In this work, a series of K*_x_* Mn*_x_* Fe_2‐_ *_x_* Mo_3_O_12_ (0.0 ≤ *x* ≤ 1.1) materials were synthesized via the solid‐state reaction. Systematic investigation of four representative compositions (KMn4, KMn6, KMn8, and KMn10) revealed that all samples maintain a hexagonal average structure across the 100–800 K. KMn4, KMn6, and KMn8 undergo a transition from low‐temperature PTE to high‐temperature ZTE. Notably, the KMn10 exhibits exceptional ZTE behavior over the entire temperature range (100–800 K), with a linear CTE of *α_l_* = 0.51 × 10^−6^ K^−1^. The reason for the distinction in low‐temperature thermal expansion between KMn4 and KMn10 was revealed through NPDF. The average and local structures of KMn10 are both hexagonal, while KMn4 exhibits local monoclinic phase distortion at low temperatures. It is the monoclinic phase distortion that causes KMn4 to exhibit PTE at low temperatures. DFT calculations and ELF analysis further demonstrated that the local distortion in KMn4 reduces structural flexibility. The analysis of Raman modes, bond lengths, and angles also indicates the presence of local structural distortions in KMn4. After crossing the local structure transition temperature, the PTE effect caused by local structure distortion disappears, and ZTE appears. Additionally, the KMn10 sample demonstrates promising infrared optical properties, with low emissivity in the near‐infrared (1–2.5 µm) band and high emissivity in the mid‐infrared (8–14 µm) band. This combination supports effective thermal management by minimizing heat absorption and facilitating radiative cooling. This work highlights the crucial role of local structure in governing macroscopic thermal expansion and presents an effective strategy for designing materials with tailored thermal expansion properties. The integration of ZTE with favorable infrared characteristics in KMn10 opens avenues for advanced applications in optics, aerospace, and energy systems, where thermal and dimensional stability are paramount.

## 4 Experiment and Characterization

### 4.1 Experiment

K*_x_* Mn*_x_* Fe_2‐_ *_x_* Mo_3_O_12_ (0 ≤ *x* ≤ 1.1) ceramics were synthesized via solid‐state reaction. Stoichiometric amounts of high‐purity raw materials: K_2_CO_3_ (West Asia, 99%), MnO (Aladdin, 99%), Fe_2_O_3_ (West Asia, 99.9%), and MoO_3_ (West Asia, 99.95%) were thoroughly mixed in an agate mortar. Ethanol was added as a grinding aid, and the mixture was ground for 2 h. This grinding process was repeated twice to ensure homogeneity and fine particle size. The mixed powder was then transferred to an alumina crucible and pre‐sintered at 600 °C for 6 h in a muffle furnace to decompose K_2_CO_3_ and release CO_2_ gas. After pre‐sintering, the powder was reground in an agate mortar for another 1 h. The resulting fine powder was uniaxially pressed into cylindrical green bodies (Φ8 × 5 mm) under 4 MPa pressure. Finally, the samples were sintered at 953 K for 10 h in a muffle furnace and cooled naturally to room temperature to obtain the target ceramics.

### 4.2 Characterization

The crystal structure information was obtained through SXRD (SPring‐8, Japan, λ = 0.6524 Å). The samples were loaded into Quartz capillary tubes. A 2D detector (XRD3025) was used to collect powder diffraction data. Automated data collection was carried out with temperature control using LabVIEWTM software along with high and low temperature nitrogen gas flow systems. The set temperature range was from 100 to 800 K, a temperature rate of 30 K/min, and a thermal equilibration delay of 3 min. Temperature‐dependent Raman spectra were collected by a Horiba LabRAM HR Evolution spectrometer in the range of 100–700 K using a laser excitation wavelength of 633 nm. X‐ray photoelectron spectroscopy (XPS, Escalab 250Xi) measurements were performed with Al Kα radiation and calibrated using the C 1s peak (284.8 eV). The NPDF data were collected at the MPI beamline of the China Spallation Neutron Source (CSNS). The IR performance of the specimen was evaluated by testing the absorptivity in the 1–2.5 µm band and the emissivity in the 3–14 µm band via an emissivity tester (IS50, ThermoScientificNicolet, USA).

### 4.3 Computational Detail

All electronic property calculations were conducted using density functional theory (DFT) within the framework of first principles, as implemented in the Vienna ab initio simulation package (VASP) [62]. The ion‐electron interactions were modeled using the projector augmented wave (PAW) method [63]. Exchange and correlation effects were accounted for via the generalized gradient approximation (GGA) with the Perdew–Burke–Ernzerhof (PBE) parametrization [64]. For the four materials, the wave functions of the unit cell were expanded in a plane‐wave basis set with an energy cutoff of 500 eV. The Brillouin zone integration was sampled using a 5 × 5 × 5 k‐point mesh, automatically generated by the Monkhorst–Pack scheme [65]. To achieve convergence to a stable crystal structure, the total energy calculations were performed with high precision, converging to 10^−8^ eV/atom, with structural relaxation ceasing when the residual forces were below 10^−4^ eV/ atom. The electron localization function (ELF) was calculated using the virtual crystal approximation (VCA) method based on the experimentally refined structures at different temperatures [66].

## Conflicts of Interest

The authors declare no conflicts of interest.

## Supporting information




**Supporting File**: advs75304‐sup‐0001‐SuppMat.docx.

## Data Availability

The data that support the findings of this study are available from the corresponding author upon reasonable request.
