# Supplementary material for: Zero Thermal Expansion and Local Structure in K x Mn x Fe2‐ x Mo3O12‐Based Materials
Source: Adv Sci (Weinh). 2026 Apr 16;13(38):e75304. doi: 10.1002/advs.75304 (PMC13335543; doi:10.1002/advs.75304)
Supplement: Supplementary file 1 — Supporting File: advs75304‐sup‐0001‐SuppMat.docx. [file ADVS-13-e75304-s001.docx]

**Supplementary Information**

**Zero Thermal Expansion and Local Structure in KMnFeMo_3_O_12_-Based Materials**

Gongsen He^1#^, Yongqiang Qiao^2#*^, Shibo Zhao^2^, Xin Chen^2^, Xiangkai Hao^2^, Kaiyue Zhao^2^, Xinglai Yuan^1^, Mengru Li^1^, Wen Yin^3^, Shintaro Kobayashi^4^, Shogo Kawaguchi^4^, Bingbing Fan^1*^, Rui Zhang^1^, Qilong Gao^2*^

1. School of Materials Science and Engineering, Zhengzhou University, Zhengzhou, 450001, China

2. Key Laboratory of Materials Physics, Ministry of Education, School of Physics, Zhengzhou University, Zhengzhou 450001, China

3. Spallation Neutron Source Science Center, Dongguan, 523803, China

4. Japan Synchrotron Radiation Research Institute (JASRI), Sayo-cho, Hyogo 679-5198, Japan

^#^These authors contributed equally to this work

Correspondence and requests for materials should be addressed to Yongqiang Qiao, Bingbing Fan, and Qilong Gao (email: [yongqiangqiao@zzu.edu.cn](mailto:yongqiangqiao@zzu.edu.cn), [fanbingbing@zzu.edu.cn](mailto:fanbingbing@zzu.edu.cn), and [qilonggao@zzu.edu.cn](mailto:qilonggao@zzu.edu.cn)).

**Figures**

**
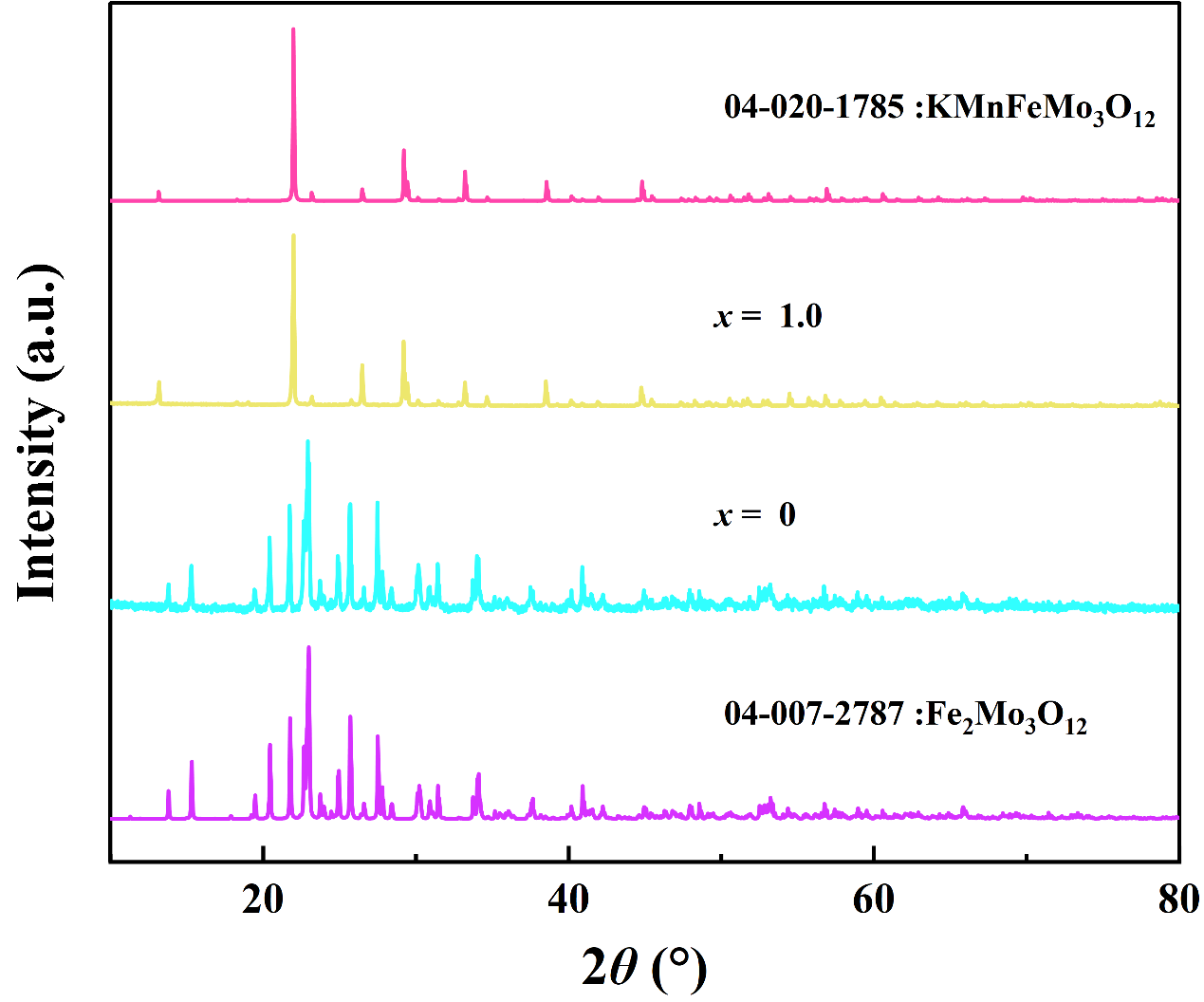
**

**Figure S1.** The XRD patterns of K*_x_*Mn*_x_*Fe_2-_*_x_*Mo_3_O_12_ (*x* = 0, 1.0) and their comparison with the standard cards.


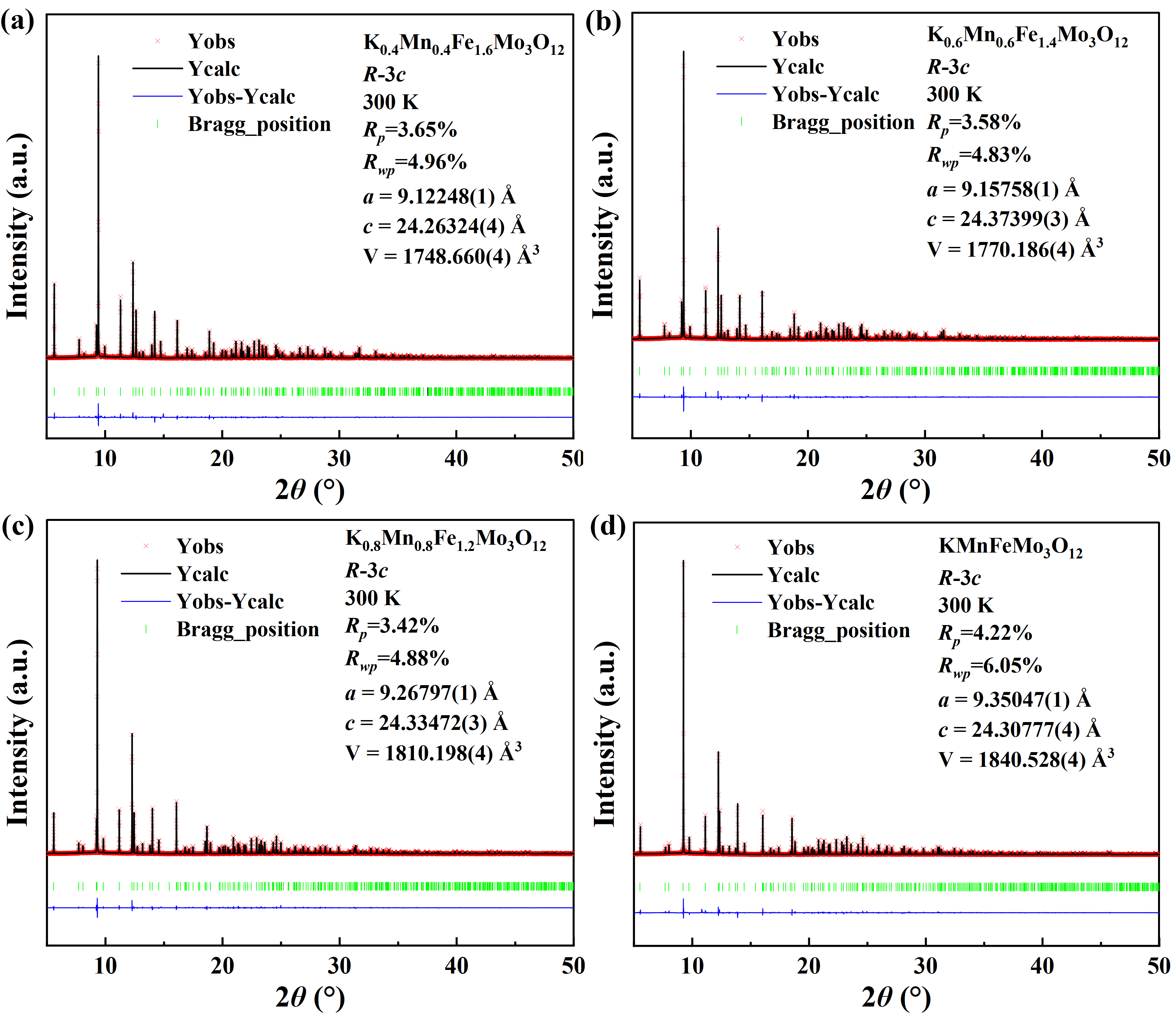


**Figure S2.** Rietveld refinement of the SXRD pattern at 300K of (a) KMn4, (b) KMn6, (c) KMn8, and (d) KMn10.


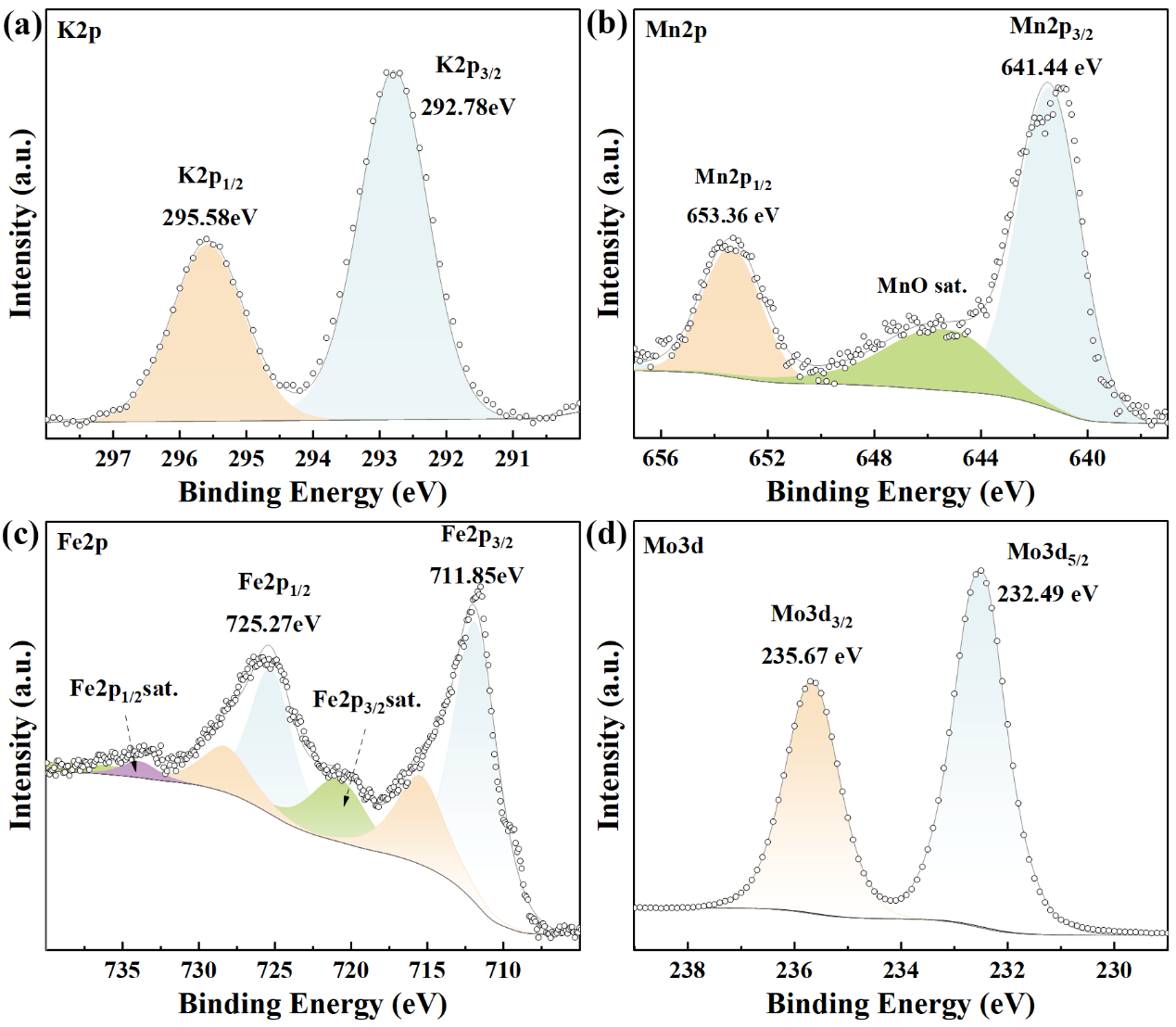


**Figure S3.** The XPS spectra of KMn4, (a) K^+^, (b) Mn^2+^, (c) Fe^3+^, and (d) Mo^6+^.

**
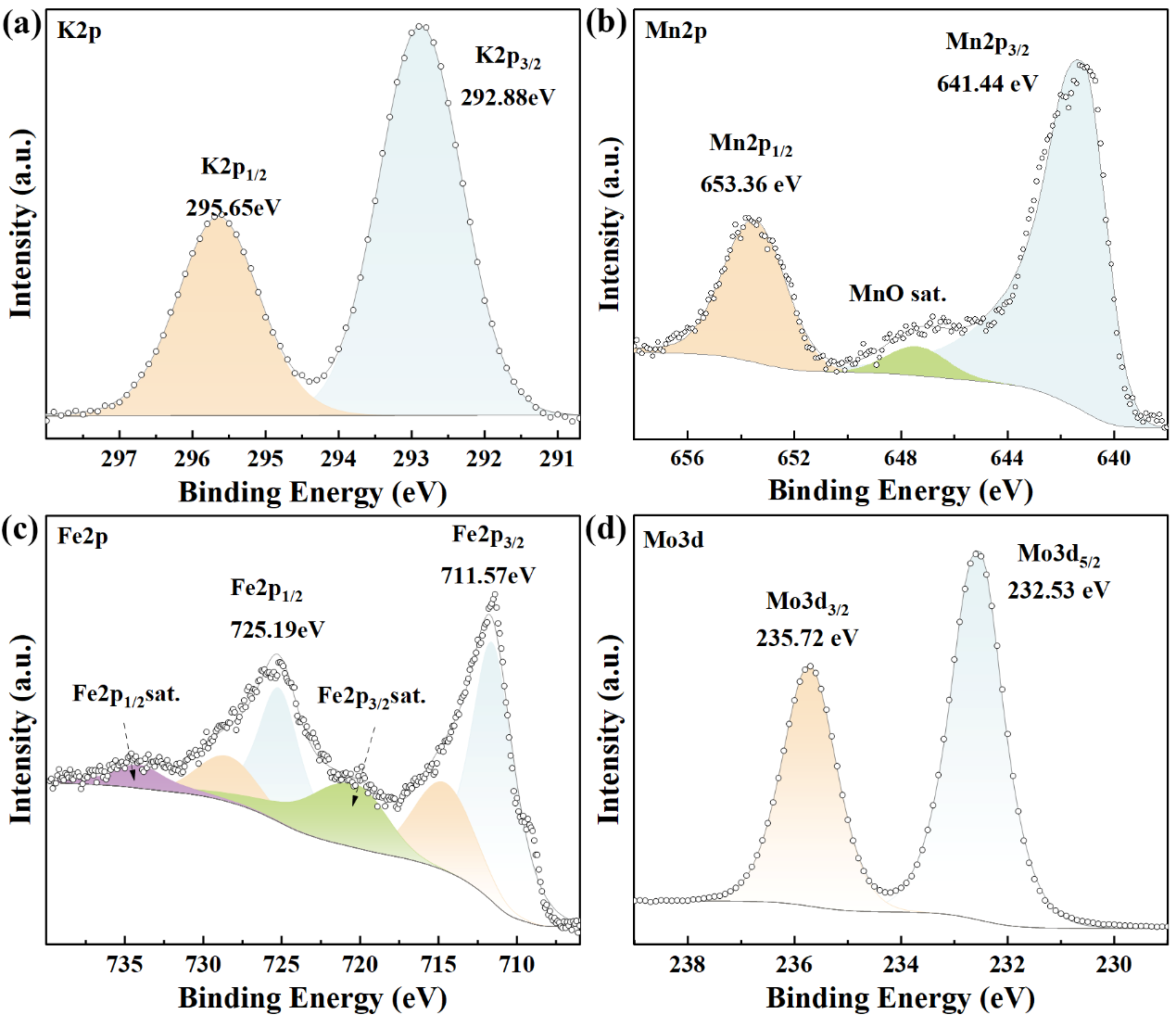
**

**Figure S4.** The XPS spectra of KMn6, (a) K^+^, (b) Mn^2+^, (c) Fe^3+^, and (d) Mo^6+^.

**
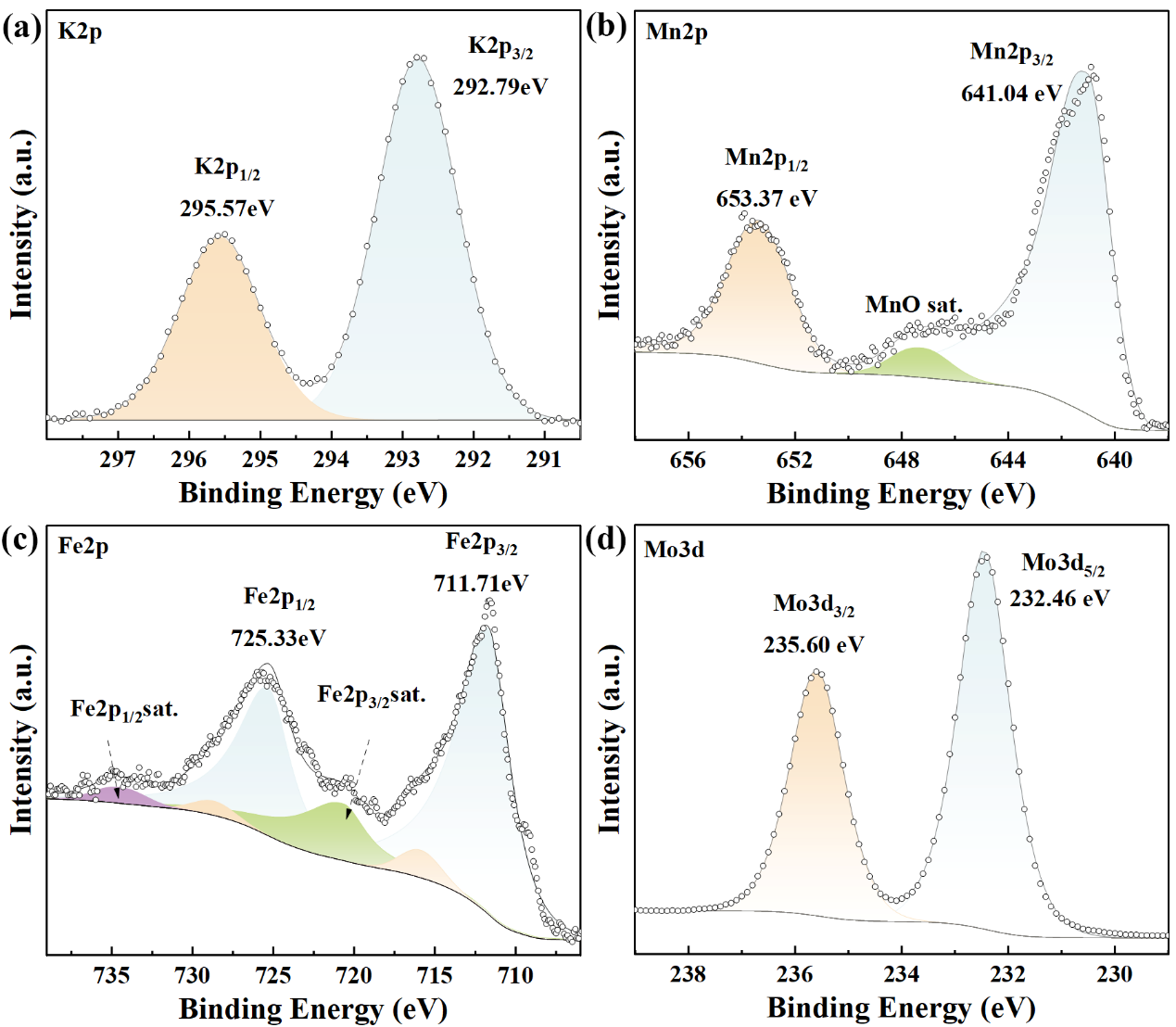
**

**Figure S5.** The XPS spectra of KMn8, (a) K^+^, (b) Mn^2+^, (c) Fe^3+^, and (d) Mo^6+^.

**
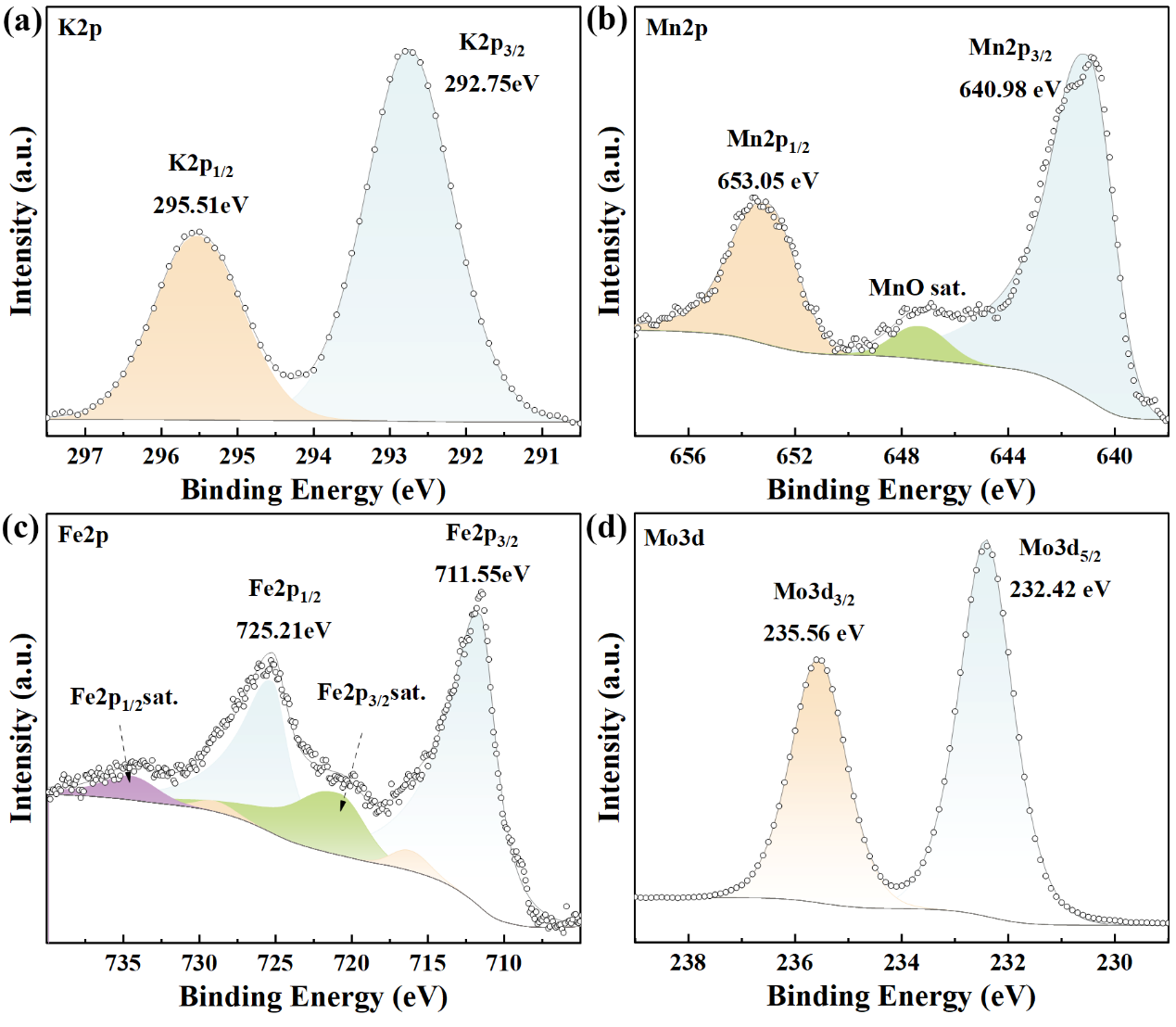
**

**Figure S6.** The XPS spectra of KMn10, (a) K^+^, (b) Mn^2+^, (c) Fe^3+^, and (d) Mo^6+^.


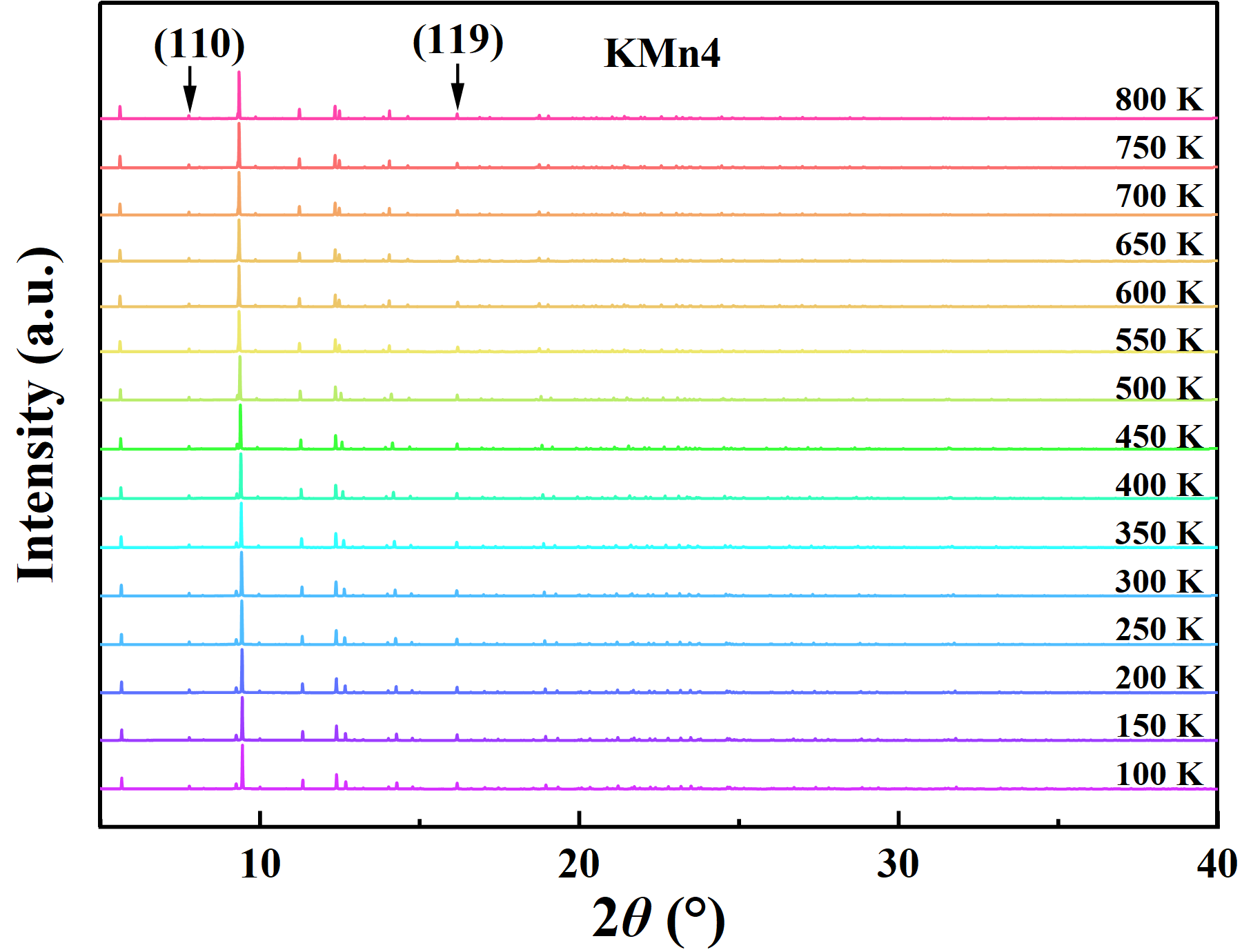


**Figure S7.** SXRD image of the KMn4 sample in the temperature range of 100 ~ 800 K.


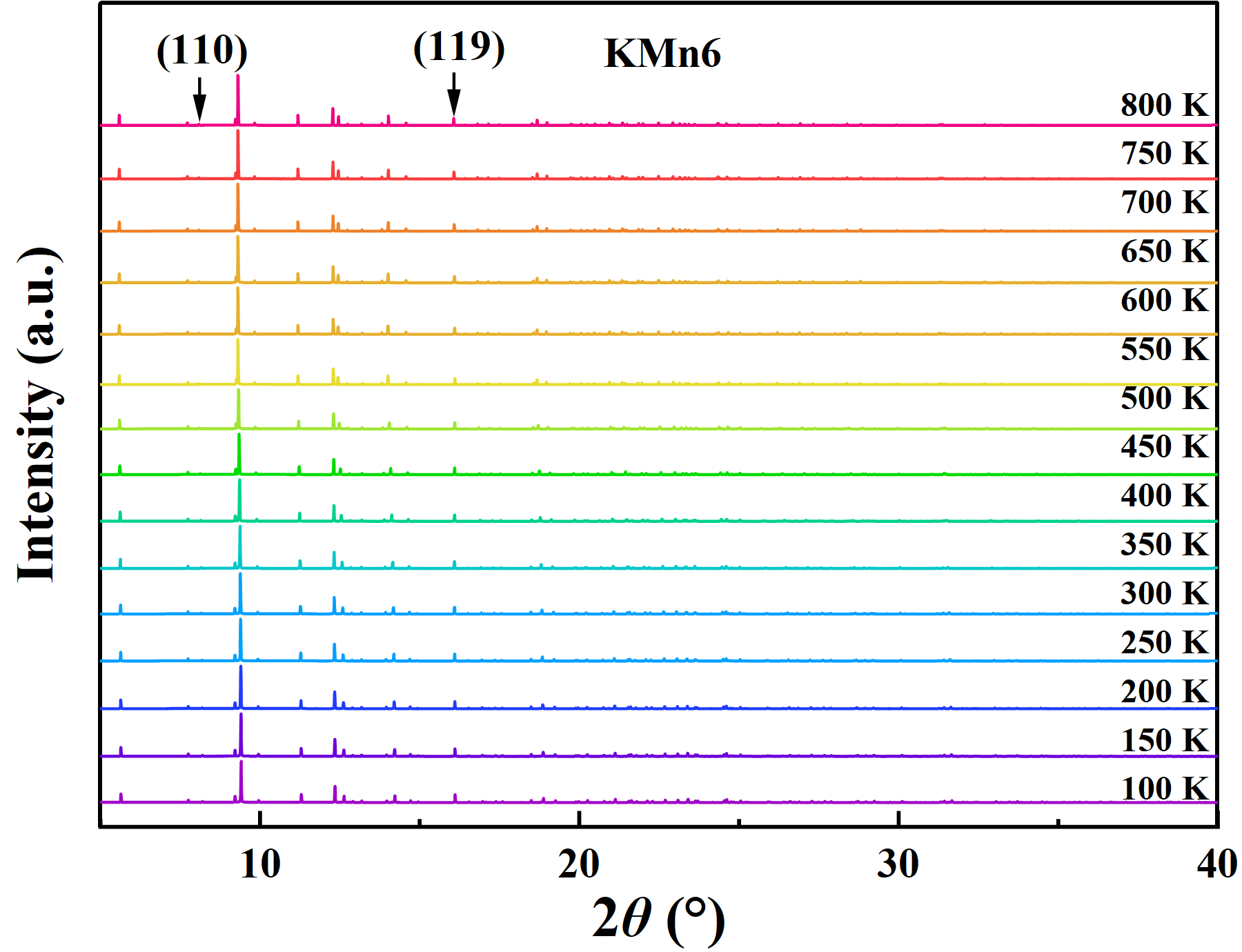


**Figure S8.** SXRD image of the KMn6 sample in the temperature range of 100 ~ 800 K.


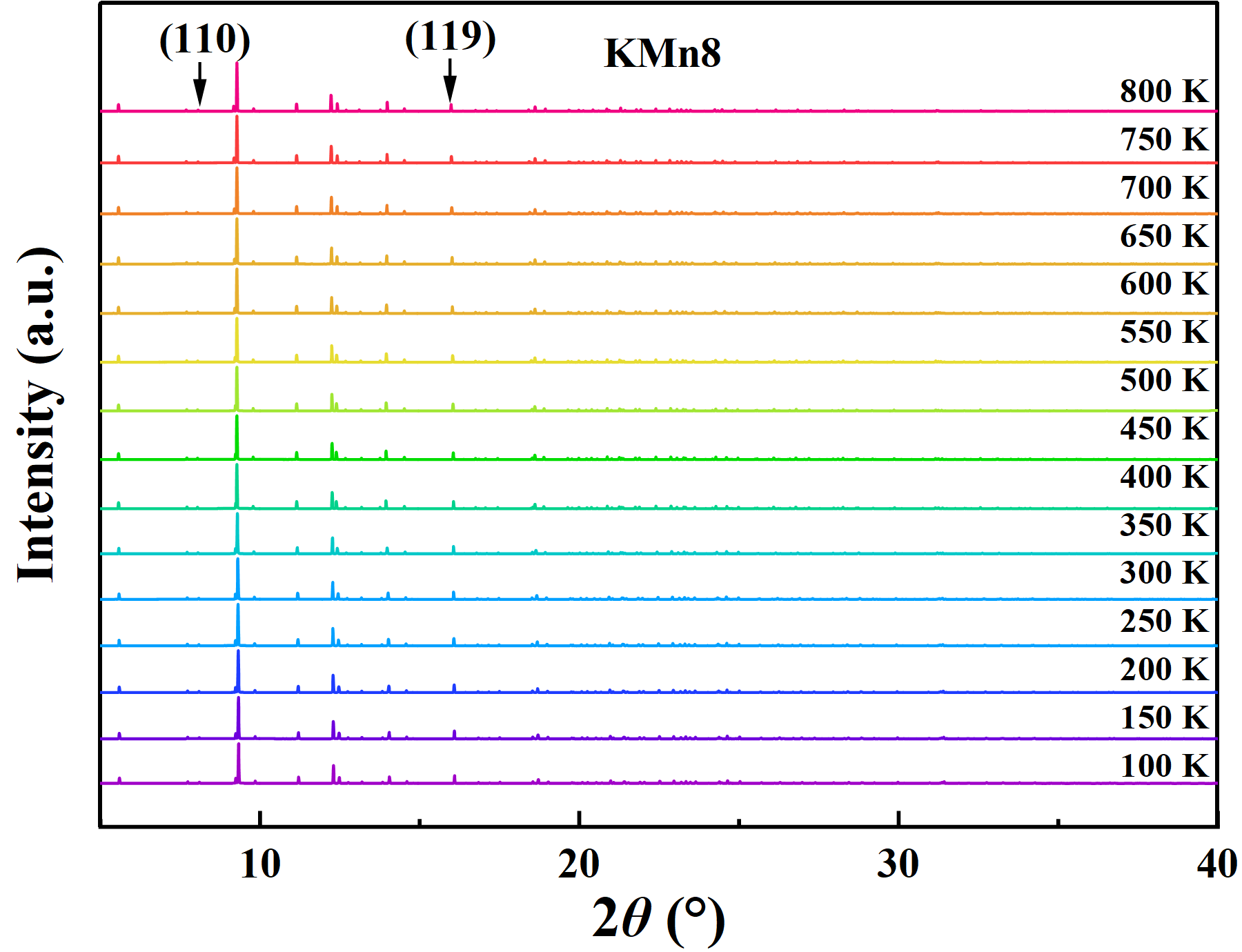


**Figure S9.** SXRD image of the KMn8 sample in the temperature range of 100 ~ 800K.


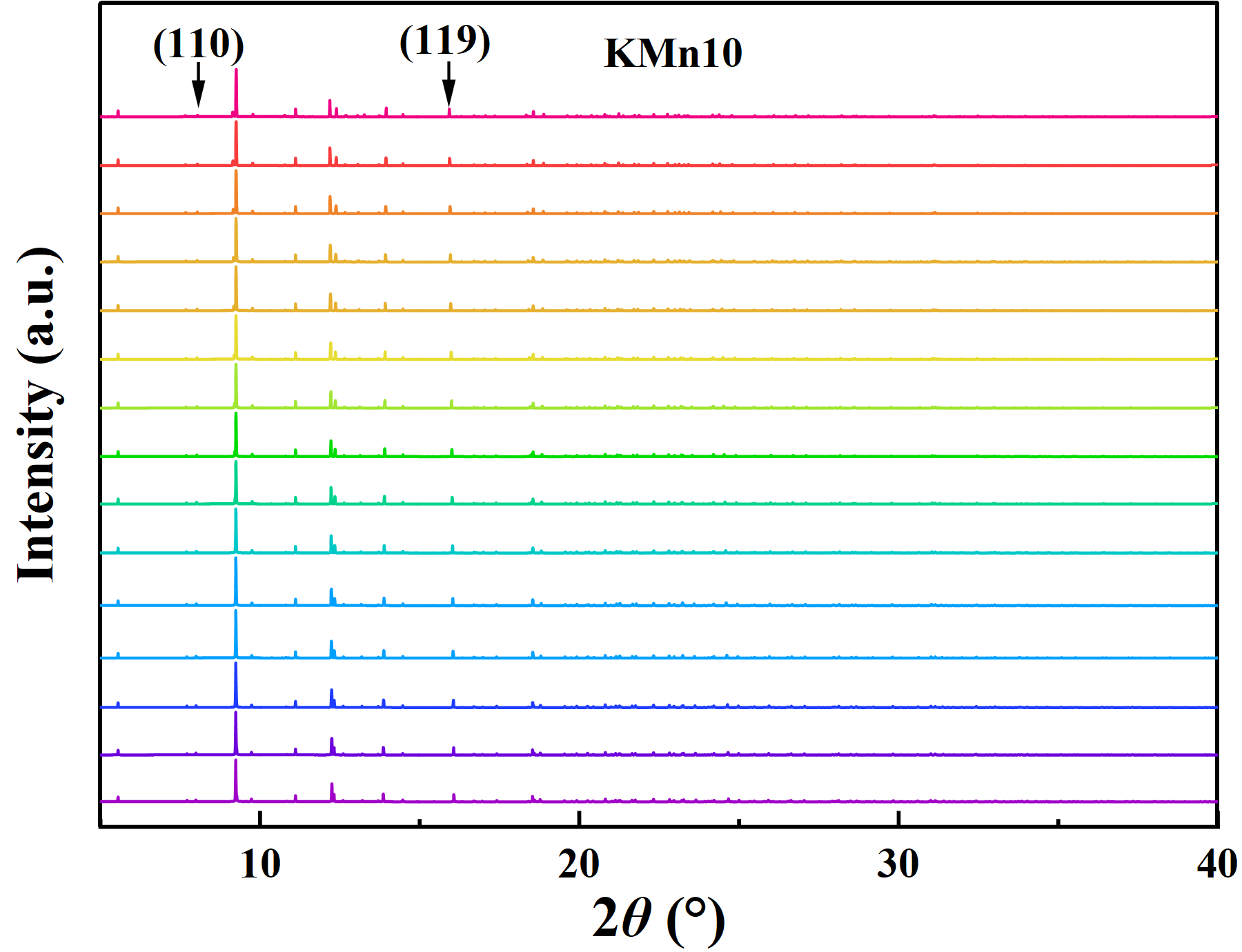


**Figure S10.** SXRD image of the KMn10 sample in the temperature range of 100 ~ 800K.


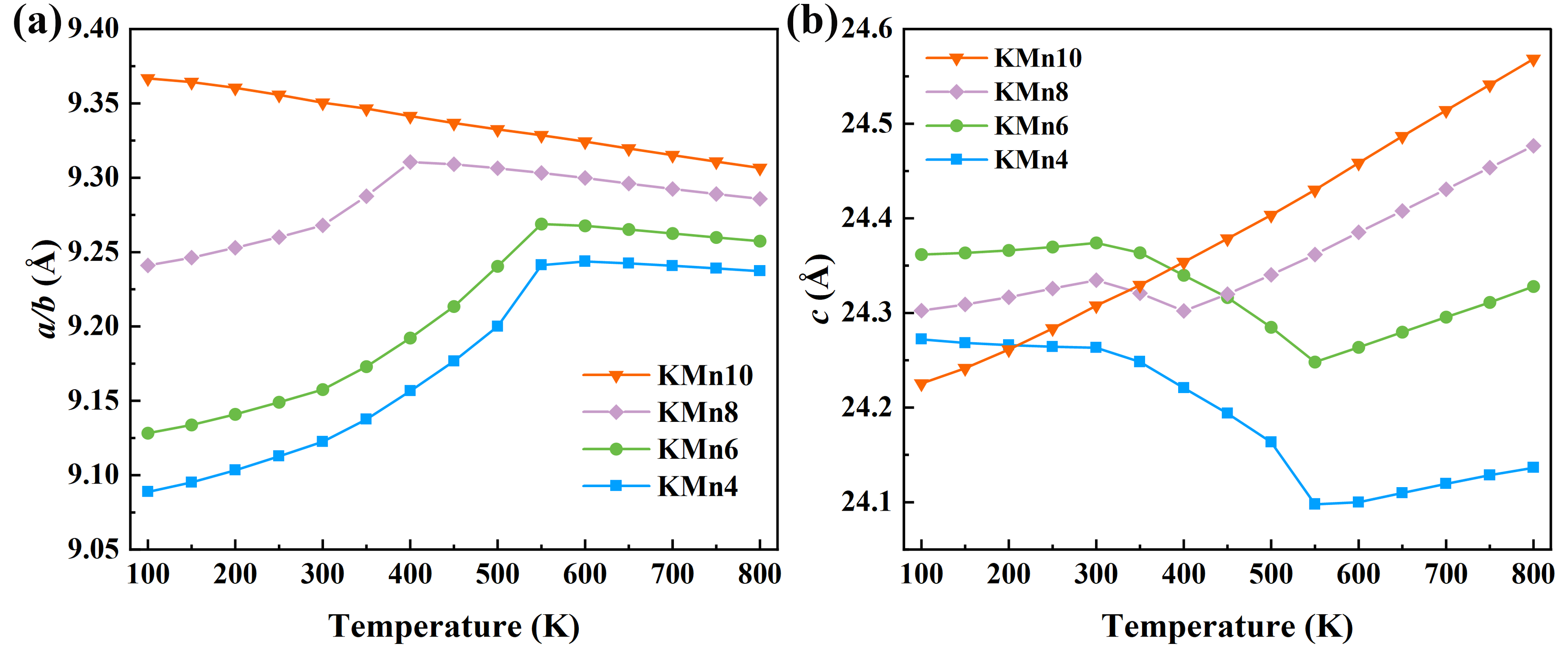


**Figure S11.** Temperature dependence of lattice parameters for (a) *a/b* axes, (b) *c* axis.

**
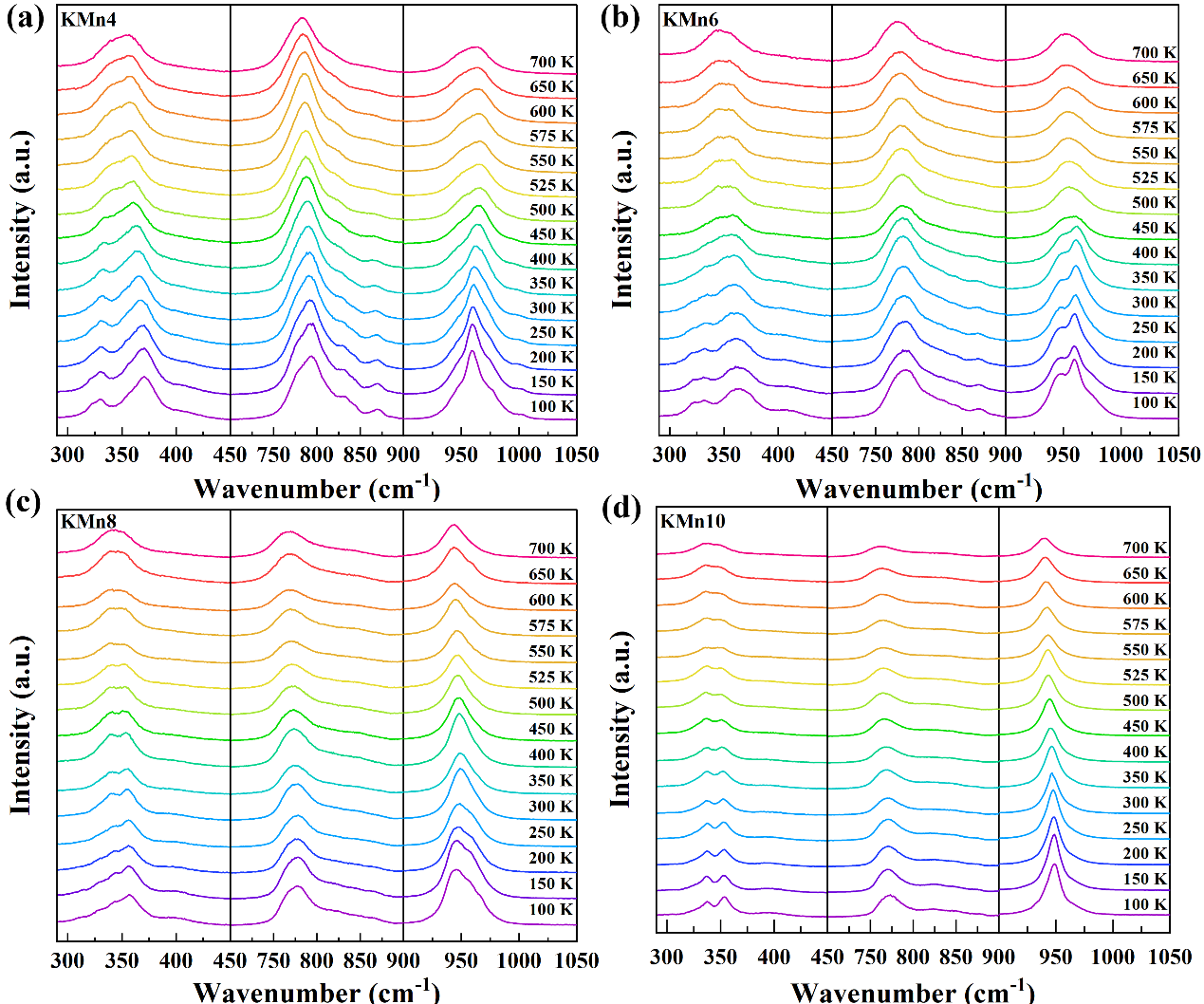
Figure S12.** (a) The temperature-dependent Raman spectra of KMn4, KMn6, KMn8, and KMn10.


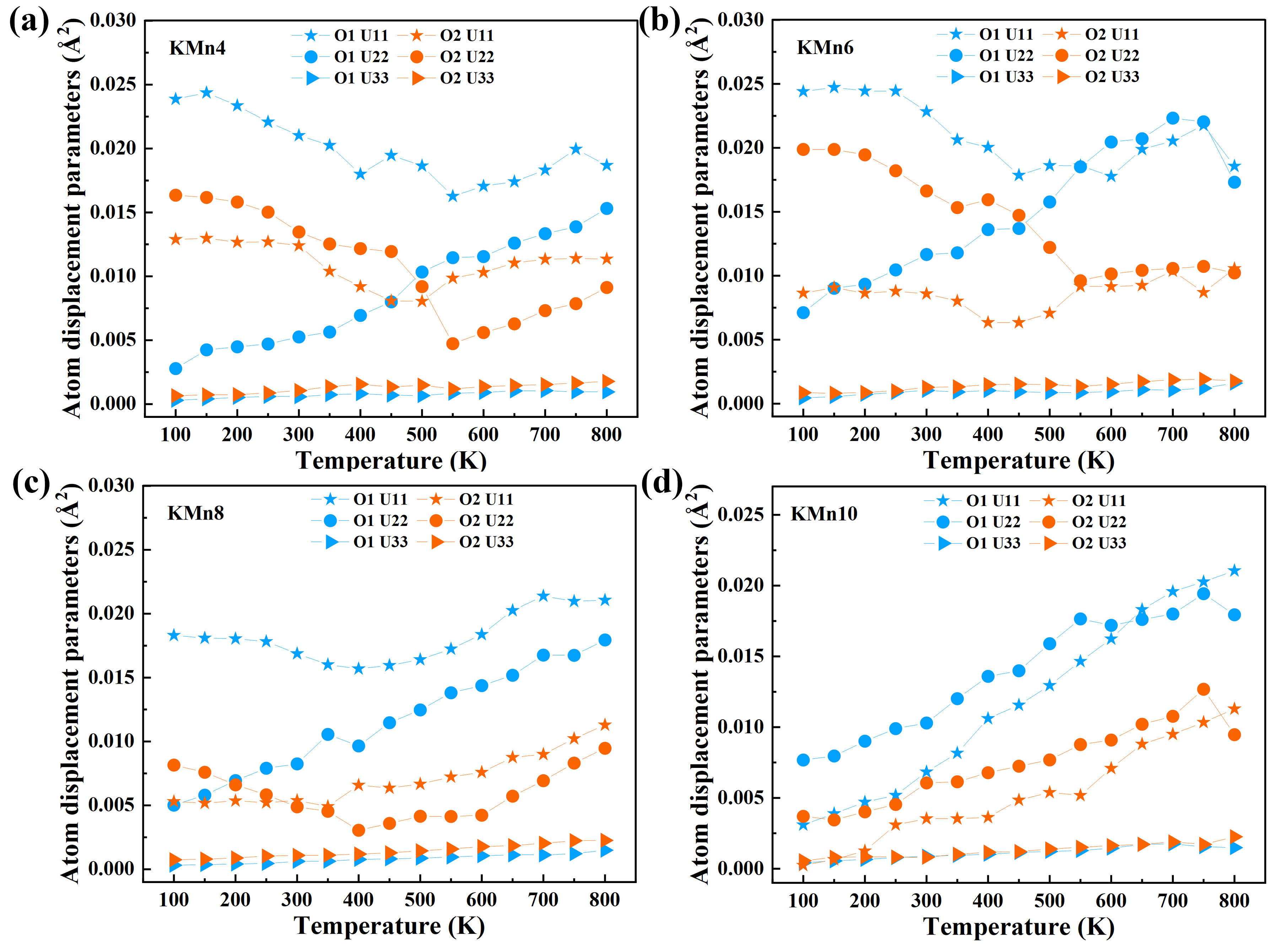


**Figure S13.** Temperature dependence of the ADPs of O atoms in (a) KMn4, (b) KMn6, (c) KMn8, and (d) KMn10.

**Tables**

**Table S1.** The optimal Rietveld refined structure parameters of KMn4 at 300 K.

| **Atom** | **Occ** | ***x*** | ***y*** | ***z*** | **Biso** |
| --- | --- | --- | --- | --- | --- |
| K | 0.4 | 0 | 0 | 0 | 3.335(63) |
| Mn | 0.4 | 0 | 0 | 0.14354(1) | 0.180(7) |
| Fe | 1.6 | 0 | 0 | 0.14354(1) | 0.180(7) |
| Mo | 3 | 0.28133(2) | 0 | 0.25 | 0.502(4) |
| O1 | 6 | 0.04280(14) | 0.19278(11) | 0.19189(4) | - |
| O2 | 6 | 0.19061(13) | 0.15610(2) | 0.09592(4) | - |

**Table S2.** The optimal Rietveld refined structure parameters of KMn6 at 300 K.

| **Atom** | **Occ** | ***x*** | ***y*** | ***z*** | **Biso** |
| --- | --- | --- | --- | --- | --- |
| K | 0.6 | 0 | 0 | 0 | 3.200(42) |
| Mn | 0.6 | 0 | 0 | 0.14379(1) | 0.197(7) |
| Fe | 1.4 | 0 | 0 | 0.14379(1) | 0.197(7) |
| Mo | 3 | 0.28159(2) | 0 | 0.25 | 0.616(4) |
| O1 | 6 | 0.03885(15) | 0.19347(13) | 0.19247(4) | - |
| O2 | 6 | 0.18922(13) | 0.15745(2) | 0.09439(4) | - |

**Table S3.** The optimal Rietveld refined structure parameters of KMn8 at 300 K.

| **Atom** | **Occ** | ***x*** | ***y*** | ***z*** | **Biso** |
| --- | --- | --- | --- | --- | --- |
| K | 0.8 | 0 | 0 | 0 | 3.013(10) |
| Mn | 0.8 | 0 | 0 | 0.14374(1) | 0.171(2) |
| Fe | 1.2 | 0 | 0 | 0.14374(1) | 0.171(2) |
| Mo | 3 | 0.28248(1) | 0 | 0.25 | 0.507(1) |
| O1 | 6 | 0.03407(12) | 0.19246(10) | 0.19160(3) | - |
| O2 | 6 | 0.18670(10) | 0.15810(9) | 0.09377(3) | - |

**Table S4.** The optimal Rietveld refined structure parameters of KMn10 at 300 K.

| **Atom** | **Occ** | ***x*** | ***y*** | ***z*** | **Biso** |
| --- | --- | --- | --- | --- | --- |
| K | 1.0 | 0 | 0 | 0 | 4.014(33) |
| Mn | 1.0 | 0 | 0 | 0.14398(1) | 0.236(9) |
| Fe | 1.0 | 0 | 0 | 0.14398(1) | 0.236(9) |
| Mo | 3 | 0.28276(2) | 0 | 0.25 | 0.578(5) |
| O1 | 6 | 0.02986(16) | 0.19330(14) | 0.19092(5) | - |
| O2 | 6 | 0.18780(12) | 0.15876(13) | 0.09220(4) | - |

**Table S5.** The anisotropic temperature factor of sample at 300 K.

| **Compound** | **Atom** | **U11** | **U22** | **U33** | **U12** | **U13** | **U23** |
| --- | --- | --- | --- | --- | --- | --- | --- |
| KMn4 | O1 | 0.02303  (69) | 0.00561  (51) | 0.00061  (6) | 0.00921  (51) | 0.00103  (15) | -0.00044  (12) |
| KMn4 | O2 | 0.01210  (57) | 0.01541  (61) | 0.00055  (7) | -0.01121  (46) | 0.00163  (15) | -0.00180  (14) |
| KMn6 | O1 | 0.02446  (77) | 0.01193  (65) | 0.00109  (6) | 0.01404  (63) | 0.00163  (17) | -0.00010  (14) |
| KMn6 | O2 | 0.00852  (58) | 0.01805  (68) | 0.00096  (7) | -0.00777  (50) | 0.00066  (15) | -0.00210  (15) |
| KMn8 | O1 | 0.01687  (56) | 0.00825  (49) | 0.00062  (4) | 0.00833  (47) | 0.00052  (13) | -0.00110  (10) |
| KMn8 | O2 | 0.00539  (43) | 0.00489  (44) | 0.00109  (5) | -0.00702  (34) | 0.00110  (12) | -0.00067  (11) |
| KMn10 | O1 | 0.00917  (64) | 0.01111  (68) | 0.00090  (7) | 0.00829  (59) | 0.00035  (16) | -0.00015  (15) |
| KMn10 | O2 | 0.00224  (52) | 0.00721  (57) | 0.00063  (7) | -0.00242  (44) | -0.00021  (14) | -0.00097  (15) |

**Table S6.** The axial CTE table of KMn4, KMn6, KMn8, and KMn10.

| **Compounds** | ***α_a/b_* (×10^-6^ K^-1^)** | ***α_a/b_* (×10^-6^ K^-1^)** | ***α_c_* (×10^-6^ K^-1^)** | ***α_c_* (×10^-6^ K^-1^)** |
| --- | --- | --- | --- | --- |
| KMn4 | 34.94  (100 ~ 550 K) | -2.20  (550 ~ 800 K) | -14.03  (100 ~ 550 K) | 6.85  (550 ~ 800 K) |
| KMn6 | 33.46  (100 ~ 550 K) | -5.13  (550 ~ 800 K) | -9.56  (100 ~ 550 K) | 13.16  (550 ~ 800 K) |
| KMn8 | 23.65  (100 ~ 400 K) | -6.94  (400 ~ 800 K) | 1.20  (100 ~ 400 K) | 18.19  (400 ~ 800 K) |
| KMn10 | -9.43  (100 ~ 800 K) | | 20.58  (100 ~ 800 K) | |

**Table S7.** CTE and temperature range of some typical ZTE materials.

| **Compound** | ***α_l_* (×10^-6^ K^-1^)** | **Temperature range (K)** | **Ref.** |
| --- | --- | --- | --- |
| Mn_3_Fe_0.2_Co_0.2_Ni_0.2_Mn_0.2_Cu_0.2_N | 0.72 | 10 ~ 180 | ^[1]^ |
| ErCo_2.8_Fe_0.2_ | 0.9 | 10 ~ 220 | ^[2]^ |
| CrVMoO_7_ | -0.64 | 100 ~ 240 | ^[3]^ |
| Hf_0.85_Ta_0.15_Fe_2_C_0.01_ | 0.8 | 85 ~ 245 | ^[4]^ |
| Sc_0.55_Ti_0.45_Fe_2_ | 0.41 | 10 ~ 250 | ^[5]^ |
| LaFe_10.1_Cu_0.5_Si_2.4_ | 0.28 | 185 ~ 250 | ^[6]^ |
| Cu_1.75_Mg_0.25_P_2_O_7_ | -0.36 | 175 ~ 253 | ^[7]^ |
| Sc_0.725_Nb_0.275_Fe_2_ | 0.69 | 108 ~ 264 | ^[8]^ |
| Zn_4_B_6_O_13_ | 1 | 13 ~ 270 | ^[9]^ |
| Gd_0.25_Dy_0.75_Co_1.93_Fe_0.07_ | 0.16 | 10 ~ 275 | ^[10]^ |
| Tb(Co_1.9_Fe_0.1_) | 0.48 | 123 ~ 307 | ^[11]^ |
| Ho_2_Fe_16_Cr | 0.43 | 13 ~ 330 | ^[12]^ |
| N(CH_3_)_4_CuZn(CN)_4_ | 0.67 | 218 ~ 368 | ^[13]^ |
| Li_0.04_(Sc_0.94_Fe_0.06_)F_3_ | -0.75 | 150 ~ 425 | ^[14]^ |
| Hf_0.6_Ti_0.4_Fe_2.5_ | 0.5 | 100 ~ 450 | ^[15]^ |
| CoHfF_6_ | 0.81 | 350 ~ 573 | ^[16]^ |
| Cs_2_W_3_O_10_ | 0.53 | 150 ~ 573 | ^[17]^ |
| Ta_2_Mo_2_O_11_ | 0.37 | 200 ~ 600 | ^[18]^ |
| Zr_0.5_Hf_0.5_VPO_7_ | 0.59 | 310 ~ 673 | ^[19]^ |
| KMn4 | 0.81 | 550 ~ 800 | This work |
| KMn6 | 0.96 | 550 ~ 800 |  |
| KMn10 | 0.51 | 100 ~ 800 |  |

**Table S8.** The values of *dω/dT* and total anharmonicity for different Raman modes of KMn4.

| **Wavenumber**  **(cm^-1^)** | ***dω*/*dT***  **(cm^-1^ K^-1^)** | **Total anharmonicity**  **(×10^-5^ K^-1^)** | **Temperature**  **(K)** |
| --- | --- | --- | --- |
| 327.8 | 0.02591 | 7.90398 | 100 ~ 700 |
| 355.8 | -0.02204 | -6.19394 | 100 ~ 525 |
| 371.1 | -0.02319 | -6.24901 | 100 ~ 700 |
| 409.8 | -0.02504 | -6.10975 | 100 ~ 700 |
| 776.0 | -0.01091 | -1.40601 | 100 ~ 700 |
| 794.3 | -0.01649 | -2.07606 | 100 ~ 700 |
| 832.3 | -0.02589 | -3.11079 | 100 ~ 700 |
| 871.3 | -0.02705 | -3.10449 | 100 ~ 700 |
| 945.3 | 0.01016 | 1.07483 | 100 ~ 500 |
|  | -0.01073 | -0.994475 | 525 ~ 700 |
| 959.5 | 0.01931 | 2.0126 | 100 ~ 500 |
|  | -0.01339 | -1.39558 | 525 ~ 700 |
| 974.6 | -0.02801 | -2.87398 | 100 ~ 350 |

**Table S9.** The values of *dω/dT* and total anharmonicity for different Raman modes of KMn6.

| **Wavenumber**  **(cm^-1^)** | ***dω*/*dT***  **(cm^-1^ K^-1^)** | **Total anharmonicity**  **(×10^-5^ K^-1^)** | **Temperature**  **(K)** |
| --- | --- | --- | --- |
| 327.1 | 0.0295 | 9.02021 | 100 ~ 700 |
| 358.7 | -0.03505 | -9.77156 | 100 ~ 525 |
| 371.7 | -0.02363 | -6.35745 | 100 ~ 700 |
| 410.1 | -0.0187 | -4.5 | 100 ~ 700 |
| 771.6 | -0.01092 | -1.41528 | 100 ~ 700 |
| 787.4 | -0.01056 | -1.34116 | 100 ~ 700 |
| 821.4 | -0.01486 | -1.80908 | 100 ~ 700 |
| 872.3 | -0.04025 | -4.61446 | 100 ~ 700 |
| 945.1 | 0.01524 | 1.61258 | 100 ~ 500 |
| 959.9 | -0.01515 | -1.60306 | 525 ~ 700 |
|  | 0.01079 | 1.12403 | 100 ~ 500 |
| 975.0 | -0.00723 | -0.753174 | 525 ~ 700 |
|  | -0.02577 | -2.64311 | 100 ~ 250 |

**Table S10.** The values of *dω/dT* and total anharmonicity for different Raman modes of KMn8.

| **Wavenumber**  **(cm^-1^)** | ***dω*/*dT***  **(cm^-1^ K^-1^)** | **Total anharmonicity**  **(×10^-5^ K^-1^)** | **Temperature**  **(K)** |
| --- | --- | --- | --- |
| 328.8 | 0.01801 | 5.47763 | 100 ~ 700 |
| 342.4 | -0.01002 | -2.92646 | 100 ~ 350 |
| 357.5 | -0.00687 | -1.92178 | 100 ~ 700 |
| 400.8 | -0.02138 | -5.33442 | 100 ~ 700 |
| 764.9 | 0.00626 | 0.818376 | 100 ~ 350 |
|  | -0.01989 | -2.60024 | 400 ~ 700 |
| 779.5 | 0.00213 | 0.273259 | 100 ~ 350 |
|  | -0.01593 | -2.04367 | 400 ~ 700 |
| 817.7 | -0.0263 | -3.21625 | 100 ~ 700 |
| 860.5 | -0.03009 | -3.49687 | 100 ~ 700 |
| 942.7 | 0.0256 | 2.71552 | 100 ~ 350 |
|  | -0.01878 | -1.99209 | 400 ~ 700 |
| 954.6 | 0.02043 | 2.14027 | 100 ~ 400 |
|  | -0.02159 | -2.26179 | 450 ~ 700 |

**Table S11.** The values of *dω/dT* and total anharmonicity for different Raman modes of KMn10.

| **Wavenumber**  **(cm^-1^)** | ***dω*/*dT***  **(cm^-1^K^-1^)** | **Total anharmonicity**  **(×10^-5^K^-1^)** | **Temperature**  **(K)** |
| --- | --- | --- | --- |
| 336.2 | -0.00133 | -0.39566 | 100 ~ 700 |
| 354.1 | -0.00497 | -1.40368 | 100 ~ 700 |
| 395.0 | -0.011 | -2.78475 | 100 ~ 700 |
| 764.9 | -0.017 | -2.22238 | 100 ~ 700 |
| 776.3 | -0.01199 | -1.54461 | 100 ~ 700 |
| 823.3 | -0.04719 | -5.73176 | 100 ~ 700 |
| 852.3 | -0.03143 | -3.68786 | 100 ~ 700 |
| 878.3 | -0.01277 | -1.45402 | 100 ~ 400 |
| 948.1 | -0.01464 | -1.54414 | 100 ~ 700 |
| 968.5 | -0.02669 | -2.7558 | 100 ~ 500 |

**Reference**

[1] Luo J, Zou K, Wang B, et al. Zero thermal expansion behavior in high-entropy anti-perovskite Mn_3_Fe_0.2_Co_0.2_Ni_0.2_Mn_0.2_Cu_0.2_N[J]. Advanced Functional Materials, 2024, 34(52): 2410608.

[2] Xu J, Wang G, Xing S, et al. Zero thermal expansion via the unconventional sublattice-magnetovolume effect in rare-earth ferrimagnets[J]. Advanced Functional Materials, 2025, 35(9): 2416314.

[3] Shi N, Kong X, Sanson A, et al. Observation of near-zero thermal expansion in CrVMoO_7_[J]. Scripta Materialia, 2023, 235: 115597.

[4] Xu J, Wang Z, Huang H, et al. Significant zero thermal expansion via enhanced magnetoelastic coupling in kagome magnets[J]. Advanced Materials, 2023, 35(8): 2208635.

[5] Song Y, Sun Q, Xu M, et al. Negative thermal expansion in (Sc, Ti)Fe_2_ induced by an unconventional magnetovolume effect[J]. Materials Horizons, 2020, 7(1): 275-281.

[6] Liu J, Gong Y, Wang J, et al. Realization of zero thermal expansion in La (Fe, Si)_13_-based system with high mechanical stability[J]. Materials & Design, 2018, 148: 71-77.

[7] Sheng B, Xie J, Shao Q, et al. Structure and abnormal thermal expansion in Cu_2-_*_x_*Mg*_x_*P_2_O_7_[J]. Physics Letters A, 2024, 525: 129843.

[8] Jing-Ting Z, Yibole H, Narsu B, et al. Structural and magnetic properties of Sc_1-_*_x_*Nb*_x_*Fe_2_ intermetallics showing anomalous zero thermal expansion[J]. Intermetallics, 2021, 136: 107252.

[9] Jiang X, Molokeev M S, Gong P, et al. Near-zero thermal expansion and high ultraviolet transparency in a borate crystal of Zn_4_B_6_O_13_[J]. Advanced Materials, 2016, 28(36): 7936-7940.

[10] Hu J, Lin K, Cao Y, et al. Adjustable magnetic phase transition inducing unusual zero thermal expansion in cubic RCo_2_-based intermetallic compounds (R = Rare Earth)[J]. Inorganic Chemistry, 2019, 58(9): 5401-5405.

[11] Song Y, Chen J, Liu X, et al. Zero thermal expansion in magnetic and metallic Tb(Co, Fe)_2_ intermetallic compounds[J]. Journal of the American Chemical Society, 2018, 140(2): 602-605.

[12] Dan S, Mukherjee S, Mazumdar C, et al. Zero thermal expansion with high Curie temperature in Ho_2_Fe_16_Cr alloy[J]. RSC Advances, 2016, 6(97): 94809-94814.

[13] Phillips A E, Halder G J, Chapman K W, et al. Zero thermal expansion in a flexible, stable framework: tetramethylammonium copper (I) zinc (II) cyanide[J]. Journal of the American Chemical Society, 2010, 132(1): 10-11.

[14] Chen J, Gao Q, Sanson A, et al. Tunable thermal expansion in framework materials through redox intercalation[J]. Nature communications, 2017, 8(1): 14441.

[15] Lin K, Zhang W, Yu C, et al. Chemical heterogeneity modulated zero thermal expansion alloy over super-wide temperature range[J]. Cell Reports Physical Science, 2023, 4(2): 101254.

[16] Qiao Y, Zhang S, Zhang P, et al. Simple chemical synthesis and isotropic negative thermal expansion in MHfF_6_ (M = Ca, Mn, Fe, and Co)[J]. Nano Research, 2024, 17(3): 2195-2203.

[17] Guo J, Fang M, Liu Q, et al. Zero thermal expansion in Cs_2_W_3_O_10_[J]. Chinese Chemical Letters, 2024, 35(7): 108957.

[18] Gao Y, Wang C, Gao Q, et al. Zero thermal expansion in Ta_2_Mo_2_O_11_ by compensation effects[J]. Inorganic Chemistry, 2020, 59(24): 18427-18431.

[19] Wang J-P, Chen Q-D, Li S-L, et al. Phase transition and near-zero thermal expansion of Zr_0.5_Hf_0.5_VPO_7_[J]. Chinese Physics B, 2018, 27(6): 066501.
